# Community health workers for non-communicable diseases prevention and control in developing countries: Evidence and implications

**DOI:** 10.1371/journal.pone.0180640

**Published:** 2017-07-13

**Authors:** Gursimer Jeet, J. S. Thakur, Shankar Prinja, Meenu Singh

**Affiliations:** 1 School of Public Health, Post Graduate Institute of Medical Education and Research, Chandigarh, India; 2 Advanced Paediatric Centre, Post Graduate Institute of Medical Education and Research, Chandigarh, India; George Institute for Global Health, INDIA

## Abstract

**Background:**

National programs for non-communicable diseases (NCD) prevention and control in different low middle income countries have a strong community component. A community health worker (CHW) delivers NCD preventive services using informational as well as behavioural approaches. Community education and interpersonal communication on lifestyle modifications is imparted with focus on primordial prevention of NCDs and screening is conducted as part of early diagnosis and management. However, the effectiveness of health promotion and screening interventions delivered through community health workers needs to be established.

**Objective:**

This review synthesised evidence on effectiveness of CHW delivered NCD primary prevention interventions in low and middle-income countries (LMICs).

**Methods:**

A systematic review of trials that utilised community health workers for primary prevention/ early detection strategy in the management of NCDs (Diabetes, cardiovascular diseases (CVD), cancers, stroke, Chronic Obstructive Pulmonary Diseases (COPD)) in LMICs was conducted. Digital databases like PubMed, EMBASE, OVID, Cochrane library, dissertation abstracts, clinical trials registry web sites of different LMIC were searched for such publications between years 2000 and 2015. We focussed on community based randomised controlled trial and cluster randomised trials without any publication language limitation. The primary outcome of review was percentage change in population with different behavioural risk factors. Additionally, mean overall changes in levels of several physical or biochemical parameters were studied as secondary outcomes. Subgroup analyses was performed by the age and sex of participants, and sensitivity analyses was conducted to assess the robustness of the findings.

**Results:**

Sixteen trials meeting the inclusion criteria were included in the review. Duration, study populations and content of interventions varied across trials. The duration of the studies ranged from mean follow up of 4 months for some risk factors to 19 months, and primary responsibilities of health workers included health promotion, treatment adherence and follow ups. Only a single trial reported all-cause mortality. The pooled effect computed indicated an increase in tobacco cessation (RR: 2.0, 95%CI: 1.11, 3.58, moderate-quality evidence) and a decrease in systolic blood pressure ((MD: -4.80, 95% CI: -8.12, -1.49, I^2^ = 93%, very low-quality evidence), diastolic blood pressure ((MD: -2.88, 95% CI: -5.65, -0.10, I^2^ = 96%, very low-quality evidence)) and blood sugar levels (glycated haemoglobin MD: -0.83%, 95%CI: -1.25,-0.41). None of the included trials reported on adverse events.

**Conclusions:**

Evidence on the implementation of primary prevention strategies using community health workers is still developing. Existing evidence suggests that, compared with standard care, using CHWs in health programmes have the potential to be effective in LMICs, particularly for tobacco cessation, blood pressure and diabetes control.

## Background

The need to address four main NCDs, i.e. cardiovascular diseases, Diabetes, chronic respiratory diseases, cancers at primary care level has become inevitable considering the rising morbidity and mortality due to this group of diseases.[1] Programmes in developing countries are being strengthened as part of a commitment towards reporting for global monitoring framework by drafting of multi-sectoral national action plans for NCDs.[2] These programmes are relying upon primary prevention as a major pillar among others.[3] In light of critical shortages in the health workforce in developing countries, the community health workers (CHWs) may serve as backbone of these primary health care services.[4] They are cost effective in comparison to other cadres of health system[5–7] and effective in delivering essential maternal & child health, family planning and nutrition health services in developing countries.[7–9] However, studies assessing their effectiveness in delivering primary prevention interventions for non-communicable disease prevention and control in LMIC settings are limited,[10] though this has been proven for developed countries.[11] Further, whatever sparse results are available are mostly through observational studies and evidence from controlled trials needs to be synthesised. Only four systematic reviews assessing this effectiveness in LMIC/ developing countries could be identified after review of published literature.[11–14] Considering this deficiency in availability of effectiveness parameters, a systematic review on effectiveness of CHW delivered primary prevention interventions for NCDs in developing countries was conducted by the authors. This was conducted following the hypothesis that for NCD control programs employing a community health worker is more likely to be successful than routine care. This manuscript details the methodologies followed and reports on findings from this systematic review.

## Methods

The doctoral research work under which this review has been conducted has received ethical approval from Institute Ethics Committee, Postgraduate Institute of Medical Education & Research, Chandigarh, India. A protocol documenting the detailed methodology of this review was registered at PROSPERO.[15] Study has been conducted and reported in line with the Preferred Reporting Items for Systematic Reviews and Meta-Analyses (PRISMA) statement guidelines (S1 PRISMA Checklist).[16]

### Description of key terms

Community health worker was defined as any health worker carrying out functions related to health care delivery; trained in some way in the context of the intervention, and having no formal professional or paraprofessional certificate or degree in tertiary education.[17] This person may deliver NCD preventive services using informational as well as behavioural approaches. The health worker may have used single component or multiple component interventions. Adult population (General/ High Risk) residing in developing countries were included as the study participants. World bank list of developing and low middle income economies was used to define the countries or regions whose data were used for effectiveness estimation.[18]

### Description of interventions to be assessed

Interventions included community health worker led health education/health promotion (life style modification advice) for diabetes, cancer, cardiovascular diseases and stroke prevention. Health topics that were studied include healthy diet, physical activity/ regular exercise, tobacco consumption/ promotion of smoking cessation, alcohol consumption, management of type 2 diabetes/ hypertension, awareness for common cancers. Studies were excluded if the intervention was not adequately described to determine that it was a CHW intervention or if the effects were not properly described to determine whether the CHWs presence produced the effects.

### Search strategy

Only randomised controlled trials (community based randomised controlled trial, cluster randomised trials) published in the last 15 years (2000–2015) were included in the review. The trial may however be started before year 2000. The time period was limited to base evidence on recently conducted studies in light of recent shift in delivery of primary health care services towards NCDs in most of the developing countries. It is worthwhile to highlight that till 2000, focus of the primary care services of most of the governments has been on maternal and child health services, family planning services, tuberculosis and HIV/AIDS.[19] It is only after release of WHO’s report highlighting lack of capacity of different LMICs for implementation of NCD prevention and control intervention in year 2000 and subsequent adoption of World Health Assembly resolution WHO 53.17 endorsing the global strategy that concrete actions started in these countries.

Due to the diversity of interventions and health topics covered, populations, study types and outcomes, a multi-stage search strategy was developed to identify relevant publications. Trials about CHWs working in NCD related promotional, preventive or curative primary healthcare in LMICs/ developing countries were included. Searches of published literature on effectiveness of NCD control interventions delivered through community health worker under the programme (focus on developing countries) were done in the following biomedical, and general reference electronic databases, without restriction to language: PubMed (2000–2015); Excerpta Medica Database (EMBASE) (2000–2015); OVID (2000–2015) and World Health Organization (WHO) library and Cochrane library. Clinical trials registers such as ClinicalTrials.gov and portals to trials registers of different developing countries through (World Health Organization (WHO) International Clinical Trials Registry Platform, ICTRP)[20] were another source of information that were reviewed for relevant literature. Website of the Health-Evidence.org was referred to for related reviews. In addition, reference lists and bibliographies of previous systematic and non- systematic reviews were screened and citation tracking were undertaken to identify relevant studies. Authors or trial investigators for further information were contacted for queries pertaining to methodology, study outcome, availability of trial reports (if results not yet published as journal article). Unpublished reports identified in a database or referenced in a publication in the initial search were also read. Abstracts and full text of identified manuscripts were reviewed. Full search strategy has been provided as supplementary material (S1 Text).

**Fig 1:**
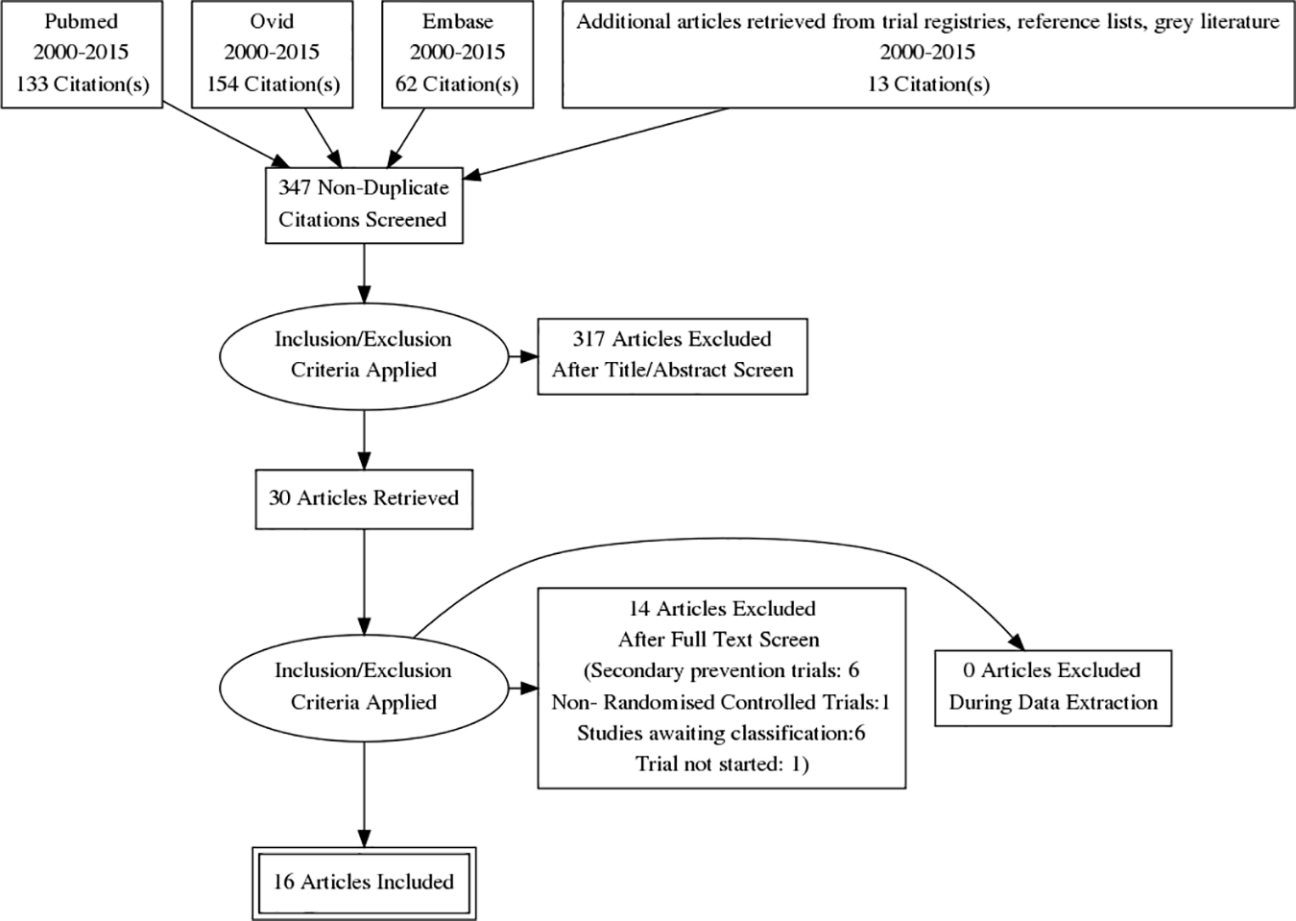
PRISMA flow diagram.

The results of the searches were entered onto the reference management software, Endnote.[21] Multiple publications of the same study identified were grouped together and represented by a single reference. Two researchers (GJ, RP) under the supervision of one supervisor (JST) independently screened the titles and abstracts identified by the electronic searches for relevancy. Titles and abstracts of all articles identified through the electronic searches were imported into EndNote and duplicates removed. A seven point inclusion criterion was followed to shortlist the articles. A research was considered eligible for inclusion if it followed a randomised controlled study design in community settings of low middle income countries with community health worker as intervention delivery person for general or high risk population for primary prevention of NCDs as compared to routine care or enhanced routine care as comparator. Full text articles were retrieved for the studies where both reviewers gave a score of 7 or ‘unclear’ to all selection criteria. Details of the flow of studies through the review are given in Figure 1 (Fig 1). The inter-rater reliability for exclusion of studies, measured as Cohen’s Kappa[22] was found to be 0.97 (SE: 0.02, 95% CI: 0.92–1.01). Disagreements during both stages were resolved by discussion or with intervention of the third reviewer (JST) who made the final decision.

### Data abstraction process

Primary reviewer (GJ) developed detailed electronic data extraction tool in MS Excel. The tool was adapted from The Cochrane Public Health Group: Data Extraction and Assessment Template to collect information on all aspects deemed necessary as per MECIR standards[23] from studies included in the review. Primarily it captured the trial’s methodological approach as well as specifics regarding how effects of the intervention were assessed. Additionally, it also collected data on methodology used for CHW selection, training, terms used to describe the CHW and their supervision. As per methodology requirements, one reviewer abstracted data (GJ) and other reviewers confirmed accuracy (JST, SP, MS). Reviewers independently dual-rated study quality and applicability using established criteria. Discrepancies were resolved through a consensus process wherein all four reviewers jointly analysed and discussed each article. Analysis was done by summarizing and discussing the data within the team. Data extraction details have been reported in registered study protocol.

**Fig 2:**
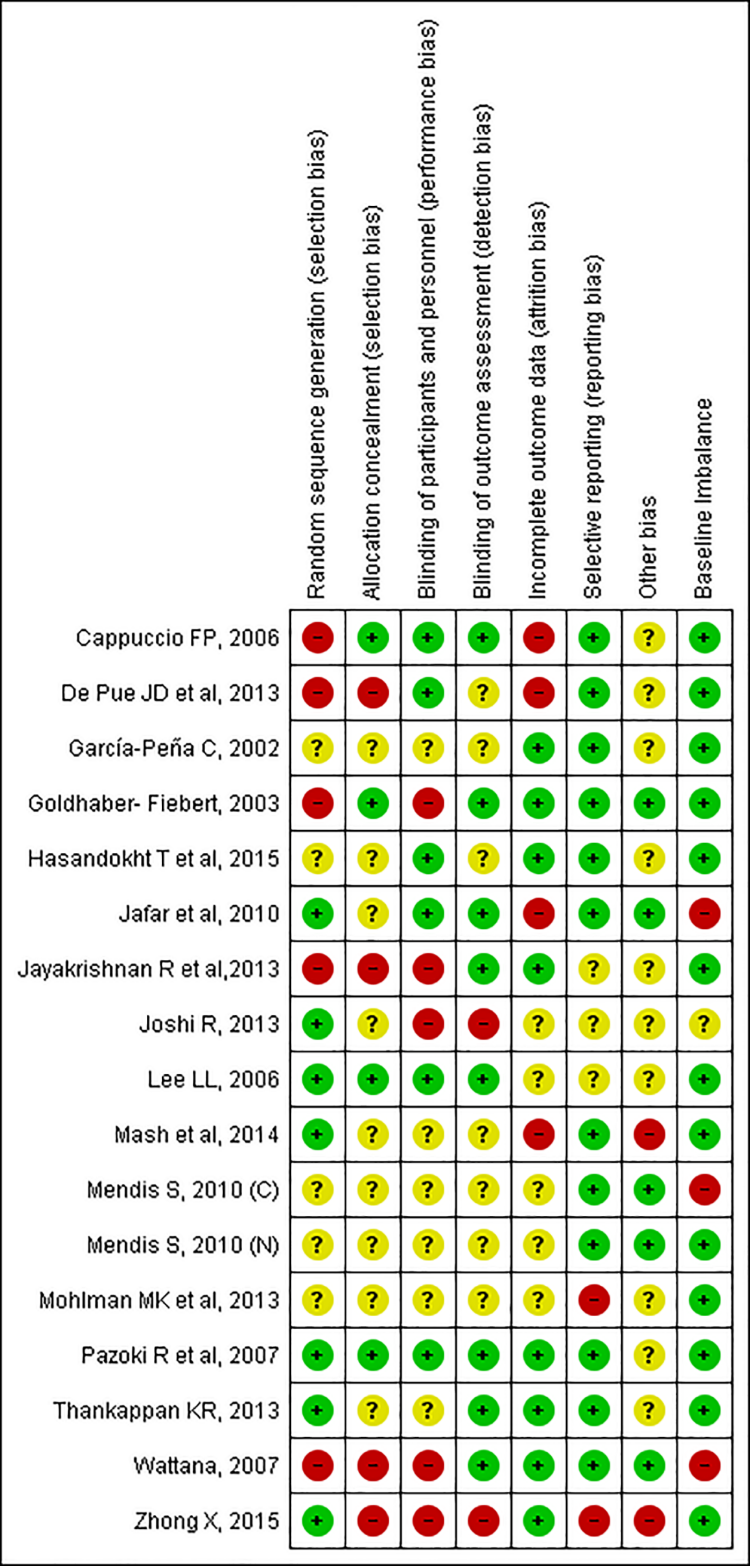
Risk of bias summary.

Quality Rating of Individual trials was done using Hamilton Effective Public Health Practice Project Checklist for quantitative studies.[24] Purpose of this quality rating was to describe the overall quality of individual studies and likelihood of bias. The quality assessment represented as global score is included in the 'Characteristics of included studies' table. (Table 1) Additionally, Cochrane risk of bias tool was used to assess risk of bias.[25] For each item, a judgement of 'High risk', 'Unclear risk', or 'Low risk' was made. Since all cluster-randomized trials randomized clusters at once, so lack of concealment of an allocation sequence was not considered as a major source of bias. Baseline comparability for individuals/clusters was reported for all the trials [26], thus reducing concern about the effects of baseline imbalance. A risk of bias summary figure has been presented in Fig 2 (Fig 2). Cohen Kappa inter-rater reliability for quality rating of the studies was found to be 0.86 (SE: 0.13, 95%CI: 0.60, 1.12).

**Table 1:** Methodological characteristics of included trials.

Study Id, Country, Setting	Disease/ Risk Factors Addressed	Population	Intervention	Control	Primary Outcomes	Global Rating
De Pue JD, 2013 American Samoa community clinics	Diabetes	High Risk (HT/ DM)	Participants were assigned to the nurse-CHW team intervention group (CHW group)	Wait list control group (usual care)	Changes in HbA1c levels	Strong
Hasandokht T, 2015 Iran Health Centres	Hypertension	High Risk (HT)	Educational lifestyle intervention program on the improvement of dietary status, physical activity level, and control of daily stress	All people over 30 years of age are screened for HTN every 3 years. Patients with high BP are visited by a physician working in rural primary care settings every 3 months but in urban primary care settings (health-care centres), patient treatment and management are passive.	Changes in systolic and diastolic blood pressure levels	Moderate
Jafar TH, 2015 Pakistan Health Centres	Hypertension,CVD, Obesity, Diabetes, Kidney disease	General Population	Home health education by community health workers alone or along with support from general practitioner	Routine care	Changes in systolic blood pressure from baseline to last follow up visit	Moderate
Mash RJ, 2014 South Africa Community (libraries, community hall)	Diabetes	High risk (DM)	Four educational group sessions lasting 20–60 minutes for a group of 15–20 people	Usual care i.e. adhoc educational talks or counselling sessions	Improved diabetes self-care activities, 5% weight loss, and a 1% reduction in HbA1c level.	Strong
Mohlman MK, 2013 Egypt community (Villages)	Tobacco	General population	The intervention consisted of a five-prong approach wherein awareness IEC/BCC activities were conducted in schools, religious institutes and finally women were sensitised regarding harms of ETS	Usual care	Prolonged Cessation	Weak
Pazoki R, 2007 Iran community	CVDs	General population	Participants received detailed program material about CVDs, risk factors of CAD, smoking and nutrition for healthy heart	Usual care i.e. no education sessions	Amount of physical activity, heart knowledge, total cholesterol	Moderate
Garcia-Pena C, 2002, Mexico households	Cardiovascular diseases	High risk (Elderly HT)	Home visits by nurses for health promotion	Usual care, i.e. routine care by family Medicine Units	Changes in systolic and diastolic blood pressure	Moderate
Thankappan KR, 2013, India community	Diabetes	High Risk	Patients in intervention group were asked and advised by a doctor to quit smoking and education materials on smoking-related complications were provided. In addition, group received four additional diabetes-specific 30-min smoking cessation counselling sessions	Usual care by physician i.e. lifestyle advice	Self-reported 7-day smoking abstinence	Moderate
Joshi R, 2012, India community	Cardiovascular diseases	General population	Received health promotion	Routine activities, i.e. no health promotion program	Mean change in knowledge score	Moderate
Jayakrishnan R, 2013, India community	Tobacco	High risk	Awareness on tobacco hazards followed by group counselling at medical camp. Individual face to face counselling sessions	Usual care i.e. routine education	Smoking abstinence	Weak
Mendis S, 2010 (C), China community	Cardiovascular diseases	High risk	WHO CVD risk management package was implemented over 4 visits	Usual care i.e. conventional treatment of hypertension	Changes in CVD risk factors; BP, Health knowledge	Moderate
Mendis S, 2010 (N), Nigeria community	Cardiovascular Diseases	High Risk	WHO CVD risk management package was implemented over 4 visits	Usual care i.e. conventional treatment of hypertension	Changes in CVD risk factors; Blood Pressure, Health Knowledge	Moderate
Lee LL, 2006, China Community	Cardiovascular Diseases	High Risk (Elderly HT)	Community based walking intervention	Usual primary health care i.e. self-initiated contact as required	Changes in systolic Blood pressure	Moderate
Goldhaber-Fiebert JD, 2003, Costa Rica Community	Diabetes	High Risk	Lifestyle intervention	Wait list control group	Changes in weight, BMI, Hba1c	Strong
Zhong X, 2015, China Community	Diabetes	General Population	Peer Leader Support Program	Wait list control group	Changes in knowledge, attitudes towards self-management, BMI	Weak
Wattana, 2007, Thailand, Community	Diabetes	High Risk Population	120 minutes small group diabetes education class, 4 small group discussions (90 minutes/group), two 45 minutes home visits by the researcher	Wait list control group	Changes in HbA1c levels	Moderate
Cappucio FP, 2006, Ghana, Community	Hypertension	General Population	Community health workers delivered sessions using flip charts as the main means of communication. These were held daily for one week and once a week thereafter, each lasting one hour (for both intervention and control arms). In addition to the standard health education package, additional advice was given to the intervention arm to limit the consumption of 5 salty foods, and when eaten, to soak the items in water overnight beforehand, and not to add salt to food.	Control villages received the standard health education package	24h urinary sodium. Systolic and diastolic blood pressure.	Weak

For trials which had not accounted for clustering, sample size estimates were adjusted for design effect using an “approximation method”.[27] Effective sample size was calculated for the comparison groups by dividing the original sample size by the design effect. Design effect was calculated as 1 + (M − 1) ICC, where M is the average cluster size and ICC is the intra-cluster correlation coefficient. If primary data was not provided, we attempted to find an appropriate ICC from the literature and adjusted the sample size accordingly.[28]

Meta-analysis was conducted using Review Manager (Revman) version 5.2.[29] Binary outcomes were reported as relative risk (RR) of outcomes in the intervention group compared to the control group. For continuous outcomes, and where baseline data were available, mean difference (MD) between the change in the intervention and control groups were reported. The outcome abstraction from the trials was not limited to the primary outcome on which the trial was based. Efforts were made to pool results for all other reported outcomes to highlight the fact that primary prevention interventions for NCDs influence several risk factors simultaneously.

Studies were pooled only if the outcomes had been measured in the same way by all studies. Where the change per group was not available [26, 30], we used end-values where randomisation was successful.[26] 95% confidence intervals (CIs) have been reported alongside all effect estimates. Meta-analyses of the correct effect estimates and standard errors from cRCTs were pooled using generic inverse-variance methods in RevMan 2012.

For interventions with multiple comparison groups, all groups that met the inclusion criteria were included in the review and meta-analyses.[31] If there were more than two relevant comparison groups we combined the relevant experimental and control groups to generate a single pair wise comparison.[31]

We encountered only one trial which followed a cross over design of RCTs.[32] However the cross-over was done after one year of intervention implementation. Therefore, we included the trial results till the end of 12 months of intervention implementation.[32] None of the included trials had unclear or missing data related to study methodology, participants’ lost to follow-up, primary outcome data, or statistical parameters, however minor queries related to data or outcome were there, for which we contacted the study's primary author via email. We recorded all missing secondary outcome data in the data extraction form and in the risk of bias table. One trial lacking baseline information collection by investigators was excluded from the meta-analysis.

Heterogeneity among the studies included in meta-analysis was assessed by visual inspection of overlap of confidence intervals, and by assessing statistical heterogeneity with the Chi^2^ statistic (P < 0.1). I^2^ statistic was computed to quantify heterogeneity; an I^2^ of 75% and above was taken as an indicator of more than desired heterogeneity. Meta-analysis was conducted only for interventions for which there were a minimum of two studies displaying sufficient homogeneity (I^2^statistic < 75%). In case of considerable heterogeneity (I^2^ > 75%) we only carried out a narrative synthesis of the results and grouped our findings by the type of outcome measured.

Meta-analyses were carried out separately for each outcome and type of study design (RCT/ cRCT). We used the random-effects model for all analyses, to incorporate any existing heterogeneity. Forest plot was generated for each comparison. In addition, we included a summary of findings table for the primary outcomes of this review (Table 2). It included the number of participants and studies for each outcome, a summary of the intervention effect, and a measure of the quality of evidence for each outcome according to GRADE considerations.

**Table 2:** Grading of evidence for different anthropometric and biochemical risk factors.

CHW led blood pressure and Diabetes control interventions for NCD prevention and control in developing countries: a systematic review of randomised controlled trials				
Patient or population: patients with NCD prevention and control in developing countries: a systematic review of randomised controlled trials				
Outcomes	Illustrative comparative risks* (95% CI)		No of Participants (studies)	Quality of the evidence (GRADE)
	Assumed risk	Corresponding risk		
	Control	Intervention		
DBP Sphygmomanometers Follow-up: mean 14 months	The mean DBP in the control groups was 84 mm Hg	The mean DBP in the intervention groups was 2.88 lower (5.65 to 0.1 lower)	6621 (11 studies)	⊕⊕⊕⊝ Moderate^1^,^2^,^3^,^4^
SBP Sphygmomanometers Follow-up: mean 14 months	The mean SBP in the control groups was 134.5 mm Hg	The mean SBP in the intervention groups was 4.8 lower (8.12 to 1.49 lower)	6782 (12 studies)	⊕⊕⊕⊝ Moderate^3^,^4^,^5^
HbA1c Biochemical methods Follow-up: mean 8 months	The mean HbA1c in the control groups was 8.80%	The mean HbA1c in the intervention groups was 0.83 lower (1.25 to 0.41 lower)	1342 (4 studies)	⊕⊕⊝⊝ Low^3^,^6^,^7^

*The basis for the **assumed risk** (e.g. the median control group risk across studies) is provided in footnotes. The **corresponding risk** (and its 95% confidence interval) is based on the assumed risk in the comparison group and the **relative effect** of the intervention (and its 95% CI). **CI:** Confidence interval

GRADE Working Group grades of evidence: **High quality:** Further research is very unlikely to change our confidence in the estimate of effect. **Moderate quality:** Further research is likely to have an important impact on our confidence in the estimate of effect and may change the estimate. **Low quality:** Further research is very likely to have an important impact on our confidence in the estimate of effect and is likely to change the estimate. **Very low quality:** We are very uncertain about the estimate.

^1^ The risk of bias summary has several unclear/ high risk fields

^2^ High heterogeneity

^3^ Small sample size large effect bias

^4^ The effect size is more than 2

^5^ No explanation was provided

^6^ Surrogate outcome for Diabetes control

^7^ Effect size between 0.5–2.0

Outcomes of interventions identified under the review were grouped into health topics identified in the protocol. In order to compare the different subgroups with each other, we conducted a standard heterogeneity test across the subgroup results, by calculating the I-square statistic. During subgroup analysis, we ensured that the subgroup data being compared were independent. In addition, we also performed a sensitivity analyses to assess the influence of study size and study design on the findings.

Detailed methodology of meta-analysis has been described in S2 Text.

### Search results

The literature search yielded 368 titles of potentially relevant articles. We identified 28 relevant articles and assessed full-text copies against the inclusion criteria. Of these, 16 RCTs finally met the inclusion criteria. One of the trial had 2 sites (Nigeria and China).[28] It was decided to analyse and present the results of both sites as different studies as there were important differences in population as well as demographic characteristics.

### Description of studies

#### Included studies

Description of study design, country, trial participants, intervention comparison group and primary outcome measures for each of the included studies in the review are given in ‘Characteristics of Included Studies’ table. (Table 1). All the studies were published between, 2002–2015. China [28, 33, 34] and India [26, 35, 36]contributed three trials each, two trials were from Iran[37] and one RCT each from American Samoa[32], Pakistan [38], South Africa[39], Mexico[40], Nigeria[28], Costa Rica[41], Thailand[42], Ghana[43]. The unit of randomisation for most of the trials (10 out of 16) was cluster randomisation.[26, 28, 32, 35, 36, 38, 39, 43–45] Remaining 6 randomised individual participants.[33, 34, 37, 40–42] Only 6 trials recruited participants from healthy population or general population. Most of the trials recruited high risk groups: known hypertensive, diabetics, elderly hypertensive. Only 4 studies were gender specific, 2 each for males and females. Percentage females’ participation in trials with both genders is 56.7%. Trials participants belonged to different ethnicities; African, Asian, American, Egyptian. For Cluster RCTs, mean cluster size ranged from 6.8–84.3.

#### Intervention providers and their training

Community health workers’ definition is very broad and depending upon availability of type of human resource the interventions get tailored. The training period varied between different trials and details of role of community health workers in different trials is given in detail in S1 Table.

#### Intervention content

Intervention content varied from trial to trial, however primarily the interventions involved interview with participant to bring about required behaviour change (S2 Table). Only those trials were included where intervention was delivered by CHW in one or more arms while routine care was given in other arm. However, in one study both intervention and control groups were reported to have attended a 4-week education programme before randomisation. The intervention group was then given additional education for 10 months.[33]

#### Excluded studies

As has been mentioned earlier, a preliminary 7 point criterion was followed to include studies in this review. However, several studies have to be excluded after preliminary inclusion. Details of these studies along with reasons for exclusion have been provided in S3 Table; Characteristics of Excluded Studies.

#### Risk of bias in included studies

**Fig 3:**
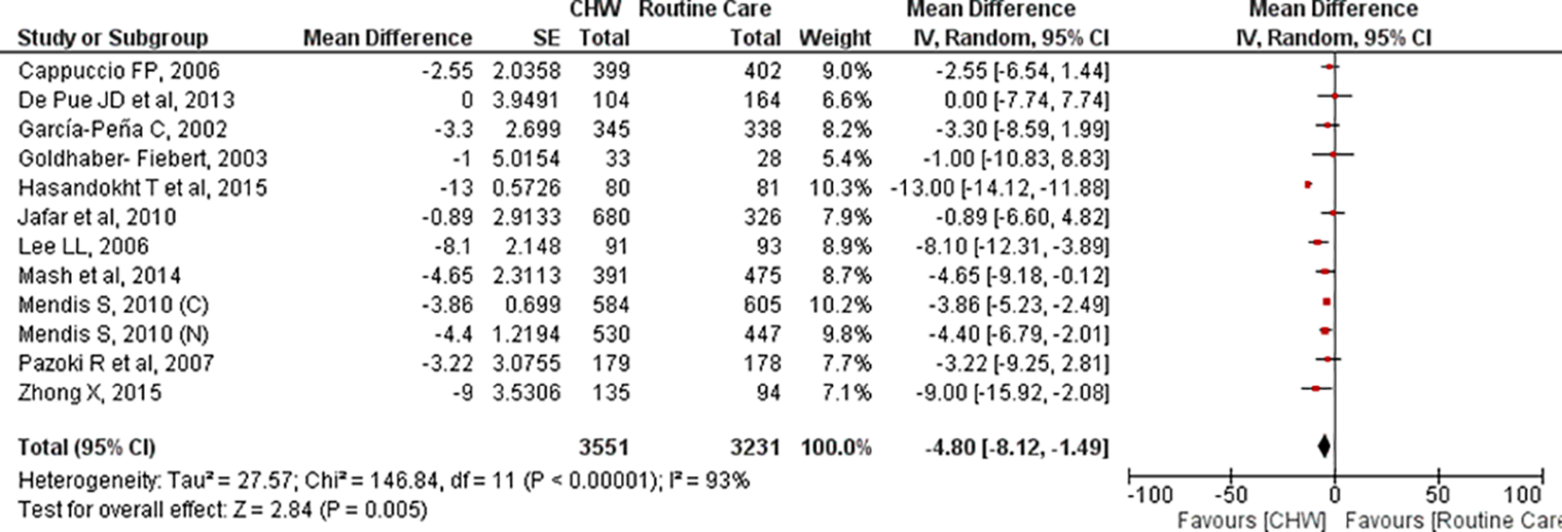
Systolic blood pressure: Mean difference.

All the studies included in the review had some source of bias. Details of risk of bias for each of the included trial have been presented in the “Risk of Bias” summary in Fig 3.

#### Diseases

16 trials included in the review were for Diabetes, Hypertension, and CVDs. No published trial addressing cancer, COPD and stroke using primary prevention strategies by CHW was found for developing countries. Trial sites of different countries, however do have ongoing registered trials. Any primary prevention trials for COPD were not found, however, in 4 trials health workers promoted tobacco cessation for control of CVD risk factors. Since “Cessation of tobacco” is the primary prevention approach to prevent COPD, we consider that the outcomes of these trials contribute indirectly to COPD prevention.

Overall the studies included in trial were at some risk of bias, and the results need careful interpretation. For comparison of quality of evidence of review, summary of findings table was generated for different risk factors using GRADE approach. Summary of review findings for different risk factors reveal varying levels of evidence, with lack of high level of evidence for any risk factor. (Table 2) We downgraded the evidence for weight and BMI by two level for very serious inconsistency because of considerable heterogeneity in effect estimates (I^2^ > 75%). Similarly, the evidence for systolic as well as diastolic blood pressure was downgraded by two level for very serious inconsistency because of considerable heterogeneity, but was upgraded by one level due to large but indirect evidence. We also downgraded the evidence for fasting blood sugar by one level for serious inconsistency because of moderate heterogeneity (I^2^ > 50%).

## Results

Most of the trials delivered primary prevention interventions as multi component interventions addressing several risk factors at the same time. Half of the total trials addressed tobacco (9 trials), blood pressure (8 trials) and physical activity (8 trials). Details of this analysis have been submitted as Supplementary material S4 Table.

### All- cause mortality

Only one trial reported all-cause mortality.[38] This trial by Jafar et al reported 67 deaths in CHW+ trained GP group, and 78 deaths in CHW group. For the purpose of review, these two groups were combined and their risk ratio was compared with the group receiving only usual care. The risk ratio for all- cause mortality in the combined group as compared to comparator group was found to be 0.91 (95% CI: 0.72, 1.17).

### Behavioural outcomes

#### Tobacco use and cessation

Out of total 17 trials, 6 trials commented upon tobacco utilisation/ cessation as an outcome. Four trials discussed past and present prevalence of tobacco use in smoking form among participants, while 3 trials reported current use of smoking. However the variables reported were different for all the trials. One trial reported change in prevalence of daily smoking.[32] Jafar et al reported current users (smoking as well as smoke less form).[38] Mash and Mohlman reported only prevalence of current smoking. All these six trials reported a decrease in prevalence of tobacco-use,[39, 44] but the cumulative risk ratio was not found to be significant (RR: 0.97, 95% CI: 0.89, 1.06). Some trials (3815 participants randomised) reported quit rates for tobacco consumption as an outcome.[28, 35, 36, 44] There was a significant difference between the intervention and control groups in the numbers of participants quitting tobacco in these studies (RR: 2.0, 95%CI: 1.11, 3.58). Overall the evidence for the CHW led interventions for tobacco use is of low quality whereas moderate for tobacco cessation (Table 3).

**Table 3:** Effectiveness estimates of reported trials for different risk factors with sensitivity of results to study or effect size.

Outcome	Studies	Participants	Statistical method	Effect estimate	Study removed (cause)	Revised effect estimate
Tobacco consumption	6	7302	RR (M-H, Random, 95% CI)	0.97 [0.89, 1.06], I^2^ = 50%	Mohlman MK (large effect, small size)	0.92 [0.89, 0.96], I^2^ = 0%
Tobacco (Quit rates)	5	2294	RR (M-H, Random, 95% CI)	2.00 [1.11, 3.58], I^2^ = 61%	Mendis S, 2010 (N) Small Effect size	2.57 [1.89, 3.50], I^2^ = 0%
Medication adherence	3	1950	RR (M-H, Random, 95% CI)	1.17 [0.98, 1.41], I^2^ = 79%	De Pue JD et al, 2013	1.07 [0.99, 1.16], I^2^ = 0%
DBP	11	6621	MD (IV, Random, 95% CI)	-2.88 [-5.65, -0.10], I^2^ = 96%	Hasandokht T et al, 2015 (large effect, small size)	-2.38 [-3.27, -1.49], I^2^ = 25%
SBP	11	6621	MD (IV, Random, 95% CI)	-4.80 [-8.12, -1.49], I^2^ = 93%	Hasandokht T et al, 2015 (large effect, small size)	-4.03 [-5.02, -3.04], I^2^ = 0%
HbA1c	4	1342	MD (IV, Random, 95% CI)	-0.83 [-1.25, -0.41], I^2^ = 0%	No change	No change
Fasting blood sugar	3	647	MD (IV, Random, 95% CI)	-1.31 [-6.42, 3.81], I^2^ = 50%	Pazoki R et al, 2007 (large effect)	-1.18 [-1.77, -0.60], I^2^ = 0%
Weight	3	1610	MD (IV, Random, 95% CI)	-2.55 [-6.24, 1.14], I^2^ = 96%	Hasandokht T et al, 2015 (large effect, small size)	-1.32 [-2.38, -0.25], I^2^ = 0%
Body mass index	8	2768	MD (IV, Random, 95% CI)	-0.74 [-1.64, 0.17], I^2^ = 95%	Hasandokht T et al, 2015 Mendis S, 2010 (N) (large effect, small size)	-0.29 [-0.70, 0.13], I^2^ = 0%
Fruits consumption	2	591	RR (M-H, Random, 95% CI)	2.25 [0.47, 10.85], I^2^ = 96%	No change	No change
Vegetable consumption	2	589	RR (M-H, Fixed, 95% CI)	1.21 [0.80, 1.83], I^2^ = 96%	No change	No change
Sodium excretion	2	1484	MD (IV, Random, 95% CI)	-0.64 [-11.67, 10.39], I^2^ = 96%	No change	No change

#### Alcohol

None of the trials reported changes in prevalence of alcohol consumption.

#### Physical activity (PA)

Six trials reported outcomes on physical activity.[32, 37–40, 45] Two trials reported both mean minutes spent (continuous scale) in physical activity and proportion of participants who became moderately active (dichotomous scale) as their results, [37, 45] while two reported only proportion of participants who became moderately active.[32, 38] Trials by Mash and Garcia-Pena reported results in different units and hence were not included in analysis.[39, 40] The current study selected proportion of participants who became moderately active (MET minutes ≥ 600 minutes as lower benchmark) as a measure for physical activity. Jafar et al took ≥ 840 MET minutes as the lower benchmark for physical activity. However as their cut off was higher than our cut off, we included their results in our analysis. Still, participants in their study with MET minutes between 600 and 840 minutes might not get included in analysis. High heterogeneity was observed between the four studies selected for analysis (I^2^ = 96%), and the overall quality of evidence was very low, hence cumulated effect size was not computed.

#### Diet

One bi-centric trial reported increased fruit and vegetable consumption as an outcome.[28] Participants at Nigeria site of the study showed a significantly increased fruit consumption (RR: 4.94, 95% CI: 3.46, 7.05) after intervention as compared to a non-significant increase in China (RR: 1.03, 95% CI: 0.77, 1.39), while vegetable consumption remained insignificant at both the study sites (RR: 0.89, 95% CI: 0.54, 1.48 at Nigeria site, RR: 2.87, 95% CI: 0.99, 4.34 at China site). Pooling results of the two study sites displayed an overall non-significant increase in fruit and vegetable intake (Table 3).

Only one trial reported the effect of advice by CHW on fat and saturated fat intake. Mean difference pre- and post-intervention was not reported to be significant.[32]

Three different trials reported findings related to salt intake. The trial by Hasandokht et al used self-reported salt intake based on food record questionnaire. Trials by Garcia Pena[40] and Cappuccio[43] measured 24 hours urinary sodium excretion levels as a surrogate indicator for salt intake. All three trials reported a non-significant decrease in urinary sodium levels post-intervention. Results of trials by Garcia Pena and Cappuccio were pooled. Though the evidence had moderate heterogeneity, the results were non-significant (RR: -0.64; 95% CI:-11.67, 10.39) Overall quality of evidence was very low.

### Physical parameters

#### Hypertension

**Fig 4:**
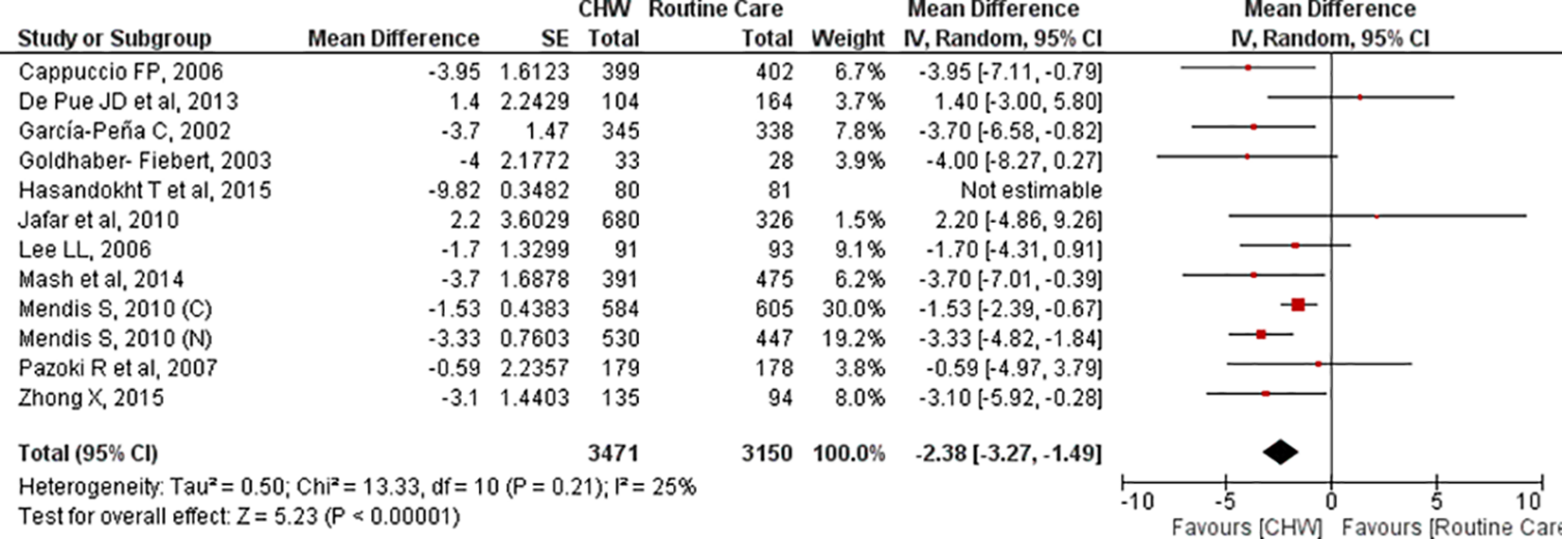
Diastolic blood pressure: Mean difference.

Systolic and diastolic blood pressure was reported by 12 trials (6782 participants randomised).[28, 32, 34, 37–41, 43, 45] The pooled effect showed a statistically significant reduction in systolic (MD: -4.80, 95% CI: -8.12, -1.49) as well as diastolic blood pressure (MD: -2.88, 95% CI: -5.65, -0.10) due to interventions. However the results needed careful interpretation due to statistically significant trial heterogeneity (SBP: I^2^ = 93%, DBP: I^2^ = 96%). (Figs 3 and 4)

On sensitivity analysis, one particular trial, by Hasandokht et al, was found to be contributing towards this heterogeneity due to its small sample size and very large effect size.[37] (Table 3) Exclusion of this trial reduced the heterogeneity in SBP and DBP to 0% and 25% respectively (MD (SBP):-4.03, 95% CI:-5.02, -3.04; MD (DBP):-2.38; 95% CI: -3.27, -1.49). The quality of evidence increased from very low in base case to moderate in the second scenario where the problematic trial was excluded.

**Fig 5:**
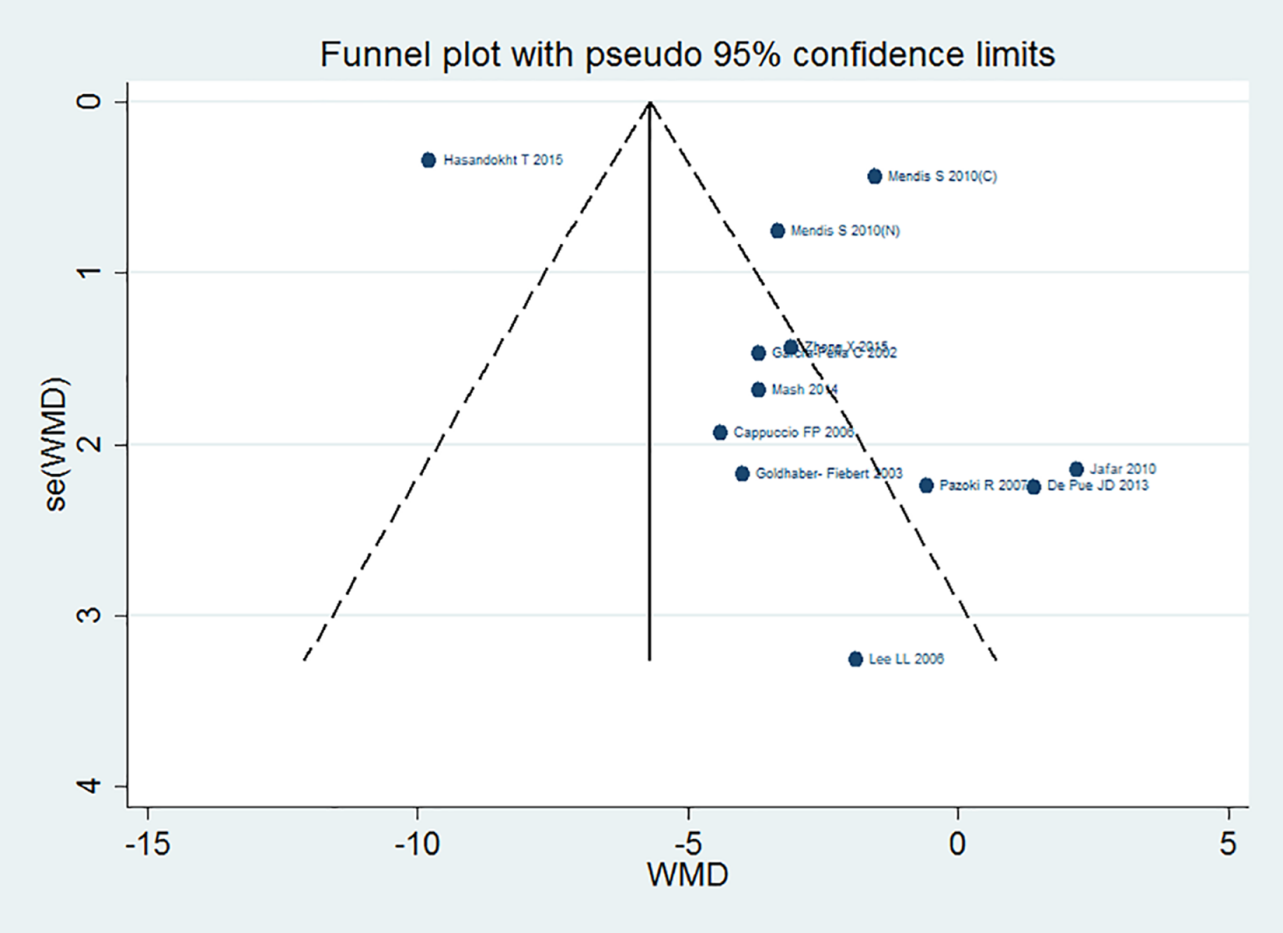
Funnel plot: Systolic blood pressure.

Likelihood of reporting bias through funnel plots was assessed only for blood pressure outcomes. There was substantial evidence of funnel plot asymmetry for both SBP and DBP, suggesting evidence of small study bias (Fig 5).

#### Weight

Four trials reported weight as an outcome.[37, 39–41] There were no statistically significant mean differences in weight between intervention and control groups from baseline in these studies. (MD: -2.55, 95% CI: -6.24, 1.14) A statistically significant trial heterogeneity (96%) was observed, which on sensitivity analysis was revealed to be because of one study[37] with highest weight and smallest sample size. Removal of this study from analysis decreased the heterogeneity to 0% and reported a statistically significant decrease in weight. (MD: -1.32, 95% CI:-2.38, -0.25). (Table 3) The quality of evidence was very low in base case scenario, which improved to low on exclusion of trial by Hasandokht from analysis.

#### Body mass index (BMI)

Eight trials reported findings on body mass index.[28, 32, 33, 37, 38, 41, 45] There were no statistically significant differences in mean BMI between intervention and control groups (MD: -0.75, 95% CI: -1.63, 0.13). Significant heterogeneity was found in these trials.

### Biochemical factors

#### Glycated haemoglobin (HbA1c)

Four trials reported glycated haemoglobin as an outcome.[32, 39, 41, 42] The pooled results presented a statistically significant difference in percentage HbA1c levels between CHW and routine care group from baseline percentage of HbA1c (MD: -0.83%, 95% CI: -1.25,-0.41).

#### Fasting blood sugar levels

Three trials reported fasting blood sugar as an outcome in their studies.[33, 41, 45] There was no statistically significant difference in mean change from baseline fasting blood glucose between the CHW and routine care group (MD: -1.31, 95% CI: -6.42, 3.81).

#### Total cholesterol

Four different trials reported on blood lipids, three on total cholesterol[39, 41, 45] and one each on triglycerides[45] and LDL[38]. There were statistically significant differences in mean change from baseline total cholesterol between intervention and control groups (MD: -0.1, 95% CI: -0.26, 0.00)

### Risk assessment

Three trials [32, 38, 42] reported risk levels of participants, however owing to different definitions of risk, the results could not be pooled into meta-analysis. Though both Wattana and Jaffar estimated CVD risk based on Framingham risk equation, Wattana reported risk as percentage, whereas Jaffar reported it as mean risk score. However risk levels improved across all the 3 trials in community health worker led interventions.

### Medication adherence

Three trials [32, 38, 40] reported medication adherence as an outcome among high risk patients for CVD development. However the trials were found to have high heterogeneity. There were no statistically significant differences between intervention and control groups in number of participants showing better medication adherence. (RR: 1.57, 95% CI: 0.95, 2.61)

### Self-efficacy

Three trials [33, 34, 39] reported changes in levels of self- efficacy for diabetes management or physical activity. However all trials had calculated efficacy on different scales; 9 item score by Zhong et al, 10 item score by Lee et al and a mean score value was given by Mash et al. Effectiveness of outcomes varied across all the 3 trials ranging from no effectiveness to significant effectiveness.

### Health care services utilisation

Only one trial[32] reported the outcomes of interventions in terms of utilisation of health care services (Primary Care Physician visits (PCP)/ Emergency department visits (ED)) before and after intervention implementation. Rate ratios (RR) for PCP visits were significantly higher in the CHW relative to the usual care group (RR = 1.71; 95% CI: 1.25, 2.33). There was no main intervention effect on ED utilization, but visits in the prior year modified the intervention effect on ED visits. Increased PCP utilization was associated with greater decreases in HbA1c (b = −0.10, se = 0.04, p = 0.01).

### Economic implications

Three trials that met the inclusion criterion, commented upon the costs or cost-effectiveness of the interventions being implemented. While one trial reported only the costs associated with intervention delivery[40], the trial by Jafar et al reported cost effectiveness for clinical outcomes only.[31] A trial from South Africa reported incremental cost effectiveness ratio (ICER) of delivering group education based intervention by health care workers as $1862/QALY gained.[46] Heterogeneity of costing methodologies, outcomes, preclude quantitative synthesis of economic estimates.

### Other outcomes

There was very limited evidence (one trial each) on outcomes such as perceived stress levels[37], depression scores[32], quality of life[42] or increase in knowledge[45]. All the trials however reported positive findings on these for the intervention group. For example, the trial by Wattana et al reported a statistically significant improvement in quality of life of patients with Diabetes after CHW led intervention (MD: 10.49; 95% CI: 0.82, 20.16). None of these trials evaluated outcomes focused on satisfaction.

## Discussion

It was predicted that NCDs will account for seven out of every ten deaths in developing countries by 2020,[47] however the rate of transition has already surpassed the prediction.[48] The rate of growth of NCDs is high and is bound to increase in coming decades. Shortfall of skilled manpower for NCD control is a reality in developing nations. With dismal doctor population ratios and complex health system contextual issues, these nations will not be able to halt NCD epidemic without involvement of health workers.[49]

Several experiences from developing as well as developed countries have been documented in last few decades related to decreased health system costs of strengthening primary care services through CHW as compared to spending on higher levels of care.[6, 50] [5, 51] Our review has also found promising evidence in favour of the effectiveness of CHWs for NCD prevention and control as compared with standard practice. An increase in favourable behavioural outcomes and decrease in most of the anthropometric (except BMI) and biochemical risk factor levels in the included studies suggest positive effect of presence of CHWs in developing settings.

Results of our review are in concordance with that of a Cochrane review by Uthman et al in 2015 which assessed the effects of multiple risk factors intervention for primary prevention of CVDs.[14] Ebrahim in 2011 had also demonstrated similar results.[52] However both these reviews did not focus on the role of CHW as a change agent. Recently two reviews focussing upon this concept have been published [12, 13] but both of these lacked quality of evidence. This adds to the importance of results of the present review.

A major strength of our study has been the methodological robustness that has been incorporated. Cochrane recommended guidelines were followed during results pooling for search strategy formulation, data abstraction and statistical analysis, in order to minimize any bias due to methodology followed. To incorporate additional quality assessment checks, EPHPP checklist was used to assess the quality of trials selected for the review in addition to use of Cochrane Risk of Bias tool. Inter-rater reliability was assessed both for exclusion of studies as well as for quality rating of studies.

A major limitation observed was the low magnitude of effect and quality of evidence in study trials included in this review. A probable reason could have been the nature of studies (community based studies, as against controlled clinical settings) which dilutes the effect of interventions due to within subject and within cluster differences. Implementation of interventions by less trained staff could also have contributed to a decrease in effect size. Most of the trials reported training the health workers in a workshop or a short course, which could also have affected the performance. With the exception of a trial by Jafar et al, all other included trials relied on short-term effects of interventions and failed to report issues related to behaviour relapse, as in interventions targeting medication adherence, weight loss or smoking. Also, shorter length of follow ups fail to capture “saturation effects” of primary prevention interventions implemented at population level.[53] However, we found that on overall synthesis of results mean follow up for most of the outcomes was more than 12 months, which adds strength to the evidence.

Minor and major methodological challenges in synthesising the evidence should also be understood while interpreting the results of this review. As we combined the results of individual and cluster randomised controlled trials, suitable analytical methods were used to make the trial results comparable. Second, the settings in which the trials were conducted varied despite developing country settings. The type of CHW, nature of interventions also were not exactly similar. The differences in study settings, baseline differences, differences in follow up periods or differences in intervention content and delivery methods led to considerable heterogeneity in evidence. We tried to address this by use of a “maximum 75% heterogeneity cut-off criteria” for selection of results of risk factors for pooling.

As countries turn towards universal healthcare coverage, policymakers will need high quality evidence about efficient strategies for NCDs, particularly those focusing on prevention, to avoid burdening health systems with large numbers of individuals with chronic conditions that are expensive to treat. Over the past decade, attention has also been increasing on the global health workforce crisis and the recognition that there will not be sufficient health manpower in virtually all countries around the world to meet the need and the demand.[54] So, in this sense, a process of task shifting will be required in order to extend services to those who need them in the face of a shortage of physicians, nurses, and other higher-level health professionals. This review fulfils a palpable deficiency in terms of evidence of CHW effectiveness from low and middle income countries. The evidence suggests that the CHWs may be effective in altering the risk factors for NCDs for people in developing countries. While community health workers’ led interventions were not very successful in altering individuals’ behaviour patterns, modifications in physical parameters, such as systolic and diastolic blood pressure was clearly observed. Community health workers were also able to introduce and sustain a long term control on HbA1C levels among diabetics, however short term effects observed were not statistically significant. It was also observed that while CHW led interventions were able to significantly reduce cholesterol levels among participants, self-efficacy and medication adherence still remain a cause of concern.

The review has also left several questions unanswered, primarily due to paucity of trials, especially in the field of cancer, COPD and stroke. This gap in information needs to be filled, which requires further research using robust trial designs and longer follow-up periods. More good quality trials need to be designed and implemented to study the effects of interventions. Measurement approaches should be made more objective and reliance on self-reported parameters should be reduced in order to enhance methodological robustness of the trials.

## Conclusion

There is limited good quality evidence from developing countries pointing towards effectiveness of primary prevention interventions targeting lifestyle factors for different NCDs. Summating existing evidence, our review establishes that community health workers led interventions have the potential to deliver NCD primary prevention interventions successfully, particularly for hypertension control, with less strong but promising indications for diabetes and body mass index. It is expected that findings of this review will help guide program and policy level decision-making of delivery of health promotion interventions to prevent rising epidemic of NCDs, thus guiding the resource allocation towards preventive and promotive services.

## Supporting information

S1 PRISMA Checklist PRISMA checklist items reported and their location within the text. (DOC)

S1 Text Search strategy. (DOCX)

S2 Text Meta-analysis details. (DOCX)

S1 Table Community health worker characteristics. (DOCX)

S2 Table Intervention contents in detail. (DOCX)

S3 Table Characteristics of excluded studies and studies awaiting classification. (DOCX)

S4 Table Matrix highlighting multicomponent nature of interventions for NCD control. (DOCX)

## Abbreviations

CHW: Community health worker
LMIC: Low- and middle-income countries
NCD: Non Communicable Disease
PRISMA: Preferred Reporting Items for Systematic Reviews and Meta-Analyses
GRADE: Grading of Recommendations, Assessment, Development and Evaluation
