# Supplementary material for: Community health workers for non-communicable diseases prevention and control in developing countries: Evidence and implications
Source: PLoS One. 2017 Jul 13;12(7):e0180640. doi: 10.1371/journal.pone.0180640 (PMC5509237; doi:10.1371/journal.pone.0180640)
Supplement: S1 Text — (DOCX) [file pone.0180640.s002.docx]

S1 Text: Search Strategy

| Search | Query |
| --- | --- |
| #1 | voluntary workers |
| #2 | voluntary worker |
| #3 | vhws |
| #4 | village health helpers |
| #5 | village drug-kit manager |
| #6 | seika |
| #7 | shasthyo karmi Schema: all |
| #8 | shasthyo karmi |
| #9 | shasthyo shebika |
| #10 | rural health motivator |
| #11 | promotora |
| #12 | outreach educator |
| #13 | mother coordinator |
| #14 | monitor |
| #15 | link worker |
| #16 | line workers |
| #17 | auxiliary |
| #18 | distributor |
| #19 | surveyor |
| #20 | assistant |
| #21 | Aide |
| #22 | health volunteer |
| #23 | community |
| #24 | community member |
| #25 | health worker |
| #26 | volunteer |
| #27 | unlicensed |
| #28 | trained |
| #29 | home health aide |
| #30 | support worker |
| #31 | allied health worker |
| #32 | allied health personnel |
| #33 | lay volunteer |
| #34 | midwives |
| #35 | midwife |
| #36 | Visit |
| #37 | Staff |
| #38 | consultant |
| #39 | caregiver |
| #40 | carer |
| #41 | helper |
| #42 | person |
| #43 | aides |
| #44 | Raise |
| #45 | Attendant |
| #46 | Visitor |
| #47 | nonprofessional workers |
| #48 | nonprofessional |
| #49 | untrained |
| #50 | LHWs |
| #51 | volunteers |
| #52 | voluntary |
| #53 | Lay |
| #54 | lady health workers |
| #55 | lady health worker |
| #56 | health extension workers |
| #57 | health extension worker |
| #58 | health visitors |
| #59 | health visitor |
| #60 | health auxiliary |
| #61 | community health workers |
| #62 | Chws |
| #63 | community resource persons |
| #64 | community worker |
| #65 | community health agent |
| #66 | community drug distributors |
| #67 | colaborador voluntary Schema: all |
| #68 | colaborador voluntary |
| #69 | community health care workers |
| #70 | community healthcare workers |
| #71 | barefoot doctor |
| #72 | brigadistas |
| #73 | basic health worker |
| #74 | ASHA |
| #75 | accredited social health activist |
| #76 | animatrice |
| #77 | anganwadi |
| #78 | agents communitaria de saude |
| #79 | agents communitaria de salud |
| #80 | activists |
| #81 | ("accredited social health activist" OR "activists" OR "agents communitaria de salud" OR "agents communitaria de saude" OR "aide" OR "aides" OR "allied health personnel" OR "allied health worker" OR "anganwadi" OR "animatrice" OR "ASHA" OR "assistant" OR "attendant" OR "auxiliary" OR "barefoot doctor" OR "basic health worker" OR "brigadistas" OR "caregiver" OR "carer" OR "chws" OR "colaborador voluntary" OR "colaborador voluntary Schema: all" OR "community" OR "community drug distributors" OR "community health agent" OR "community health care workers" OR "community health workers" OR "community healthcare workers" OR "community member" OR "community resource persons" OR "community worker" OR "consultant" OR "distributor" OR "health auxiliary" OR "health extension worker" OR "health extension workers" OR "health visitor" OR "health visitors" OR "health volunteer" OR "health worker" OR "helper" OR "home health aide" OR "lady health worker" OR "lady health workers" OR "Lay" OR "lay volunteer" OR "LHWs" OR "line workers" OR "link worker" OR "midwife" OR "midwives" OR "monitor" OR "mother coordinator" OR "nonprofessional" OR "nonprofessional workers" OR "outreach educator" OR "person" OR "promotora" OR "raise" OR "rural health motivator" OR "seika" OR "shasthyo karmi" OR "shasthyo karmi Schema: all" OR "shasthyo shebika" OR "staff" OR "support worker" OR "surveyor" OR "trained" OR "unlicensed" OR "untrained" OR "vhws" OR "village drug-kit manager" OR "village health helpers" OR "visit" OR "visitor" OR "voluntary" OR "voluntary worker" OR "voluntary workers" OR "volunteer" OR "volunteers") |
| #82 | "fast food" |
| #83 | junk food |
| #84 | walk* |
| #85 | Walking |
| #86 | Jogging |
| #87 | Running |
| #88 | exp stress/ |
| #89 | alcohol consumption |
| #90 | "alcohol" |
| #91 | smoking behaviour |
| #92 | exp smoking/ |
| #93 | " regular exercise" |
| #94 | "exercise" |
| #95 | "physical inactivity" |
| #96 | "less than five servings" |
| #97 | "Five a day" |
| #98 | "5 a day" |
| #99 | low intake |
| #100 | vegetable intake |
| #101 | vegetable intake |
| #102 | vegetable consumption |
| #103 | Vegetables |
| #104 | "fruits and vegetables" |
| #105 | Fruits |
| #106 | Fruit |
| #107 | exp food habits |
| #108 | Nutrition |
| #109 | diet* |
| #110 | "tobacco smoke" |
| #111 | Tobacco |
| #112 | exp drinking/ |
| #113 | exp alcohol drinking/ |
| #114 | waist circumference |
| #115 | Obese |
| #116 | Obesity |
| #117 | Overweight |
| #118 | "Raised BMI" |
| #119 | "Raised BMI" |
| #120 | BMI |
| #121 | Body Mass Index |
| #122 | Triglycerides |
| #123 | high triglycerides |
| #124 | raised cholesterol |
| #125 | Cholesterolemia |
| #126 | Cholesterolemia |
| #127 | cholestrol |
| #128 | "diabetes mellitus" |
| #129 | diabetes |
| #130 | raised blood pressure |
| #131 | hypertension |
| #132 | "CVD risk" |
| #133 | CVD |
| #134 | Cardiovascular diseases |
| #135 | Cardiovascular disease |
| #136 | Cancer |
| #137 | Risk Factors |
| #138 | life style factors |
| #139 | Chronic disease |
| #140 | NCDs |
| #141 | NCD |
| #142 | Noncommunicable |
| #143 | Noncommunicable Disease |
| #144 | Non communicable Disease |
| #145 | Non communicable |
| #146 | (((((((((((((((((((((((((((((((((((((((((((((((((((((((((((((((Non communicable) OR Non communicable Disease) OR Noncommunicable Disease) OR Noncommunicable) OR NCD) OR Chronic disease) OR Risk Factors) OR Cancer) OR Cardiovascular disease) OR CVD) OR "CVD risk") OR hypertension) OR life style factors) OR NCDs) OR Cardiovascular diseases) OR raised blood pressure) OR diabetes) OR "diabetes mellitus") OR cholestrol) OR cholestrolemia) OR cholesterolemia) OR raised cholesterol) OR high triglycerides) OR triglycerides) OR Body Mass Index) OR BMI) OR " Raised BMI") OR "Raised BMI") OR Overweight) OR obesity) OR obese) OR waist circumference) OR exp alcohol drinking/) OR exp drinking/) OR tobacco) OR "tobacco smoke") OR diet*) OR nutrition) OR exp food habits) OR fruit) OR fruits) OR ("fruits and vegetables")) OR vegetables) OR vegetable consumption) OR vegetable intkae) OR vegetable intake) OR low intake) OR "5 a day") OR "Five a day") OR "less than five servings") OR "physical inactivity") OR "exercise") OR " regular exercise") OR exp smoking/) OR smoking behaviour) OR "alcohol") OR alcohol consumption) OR exp stress/) OR running) OR jogging) OR walking) OR walk*) OR junk food) OR "fast food" |
| #147 | multiple risk behavior* program* |
| #148 | multiple risk behaviour* program* |
| #149 | multiple risk behavior* intervention* |
| #150 | multiple risk behaviour* intervention* |
| #151 | multiple behaviour* risk factor* program* |
| #152 | multiple behavior* risk factor* program* |
| #153 | multiple behaviour* risk factor* intervention* |
| #154 | multiple behavior* risk factor* intervention* |
| #155 | multiple health behaviour* change intervention* |
| #156 | multiple health behavior* change intervention* |
| #157 | health behaviour* intervention* |
| #158 | health behaviour* program* |
| #159 | health behavior* intervention* |
| #160 | health behavior* program* |
| #161 | multifactorial lifestyle program* |
| #162 | multifactorial lifestyle intervention* |
| #163 | multiple risk factor intervention* |
| #164 | multiple risk factor program* |
| #165 | behaviour* change program* |
| #166 | behaviour* change intervention* |
| #167 | behavior* change program |
| #168 | behavior* change intervention |
| #169 | life style intervention |
| #170 | lifestyle programs |
| #171 | lifestyle program |
| #172 | lifestyle interventions |
| #173 | lifestyle intervention |
| #174 | ((((((((((((((((((((((((((multiple risk behavior* program*) OR multiple risk behaviour* program*) OR multiple risk behavior* intervention*) OR multiple risk behaviour* intervention*) OR multiple behaviour* risk factor* program*) OR multiple behavior* risk factor* program*) OR multiple behaviour* risk factor* intervention*) OR multiple behavior* risk factor* intervention*) OR multiple health behaviour* change intervention*) OR multiple health behavior* change intervention*) OR health behaviour* intervention*) OR health behaviour* program*) OR health behavior* intervention*) OR health behavior* program*) OR multifactorial lifestyle program*) OR multifactorial lifestyle intervention*) OR multiple risk factor intervention*) OR multiple risk factor program*) OR behaviour* change program*) OR behaviour* change intervention*) OR behavior* change program) OR behavior* change intervention) OR life style intervention) OR lifestyle programs) OR lifestyle program) OR lifestyle interventions) OR lifestyle intervention |
| #175 | controlled trial |
| #176 | controlled |
| #177 | control |
| #178 | "group randomised" |
| #179 | "group randomized" |
| #180 | group randomized trial |
| #181 | cluster randomized |
| #182 | cluster randomization |
| #183 | cluster randomisation |
| #184 | interventions |
| #185 | intervention |
| #186 | random* |
| #187 | randomizes |
| #188 | randomized |
| #189 | "randomized controlled trial" |
| #190 | randomized controlled trial |
| #191 | (((((((((((((((randomized controlled trial[Publication Type]) OR "randomized controlled trial"[Publication Type]) OR randomized[Text Word]) OR randomizes[Text Word]) OR random*[Text Word]) OR intervention[Text Word]) OR interventions[Text Word]) OR cluster randomisation) OR cluster randomization) OR cluster randomized) OR group randomized trial) OR "group randomized") OR "group randomised") OR control) OR controlled) OR controlled trial |
| #192 | Rhodesia |
| #193 | Zimbabwe |
| #194 | Zambia |
| #195 | Yugoslavia |
| #196 | Yemen |
| #197 | West Bank |
| #198 | Viet Nam |
| #199 | Vietnam |
| #200 | Venezuela |
| #201 | New Hebrides |
| #202 | Hebrides |
| #203 | Vanuatu |
| #204 | Uzbek |
| #205 | Uzbekistan |
| #206 | Soviet Union |
| #207 | USSR |
| #208 | Uruguay |
| #209 | Ukraine |
| #210 | Uganda |
| #211 | Tuvalu |
| #212 | Turkmen |
| #213 | Turkmenistan |
| #214 | Turkey |
| #215 | Tunisia |
| #216 | Tobago |
| #217 | Trinidad |
| #218 | Tonga |
| #219 | Togolese Republic |
| #220 | Togo |
| #221 | Thailand |
| #222 | Tanzania |
| #223 | tadzhik |
| #224 | Tadzhikistan |
| #225 | Tajikistan |
| #226 | Syrian |
| #227 | Syria |
| #228 | Swaziland |
| #229 | Surinam |
| #230 | Suriname |
| #231 | Sudan |
| #232 | Somalia |
| #233 | Solomon Islands |
| #234 | Ceylon |
| #235 | Sri lanka |
| #236 | Slovenia |
| #237 | Sierra Leone |
| #238 | Seychelles |
| #239 | Montenegro |
| #240 | Serbia |
| #241 | Senegal |
| #242 | Saudi Arabia |
| #243 | Sao Tome |
| #244 | Navigator Island |
| #245 | Navigator Islands |
| #246 | Samoan Islands |
| #247 | Samoa |
| #248 | Grenadines |
| #249 | St Lucia |
| #250 | St Vincent |
| #251 | Saint Vincent |
| #252 | Saint Lucia |
| #253 | Nevis |
| #254 | St Kitts |
| #255 | Saint Kitts |
| #256 | Ruanda |
| #257 | Rwanda |
| #258 | Russian |
| #259 | Russia |
| #260 | Roumania |
| #261 | Rumania |
| #262 | Romania |
| #263 | Puerto Rico |
| #264 | Portugal |
| #265 | Poland |
| #266 | Philipines |
| #267 | Philipine |
| #268 | Philippine |
| #269 | Philippines |
| #270 | Peru |
| #271 | Paraguay |
| #272 | Panama |
| #273 | Palestine |
| #274 | Palau |
| #275 | Pakistan |
| #276 | Muscat |
| #277 | Oman |
| #278 | Northern Mariana Islands |
| #279 | Nigeria |
| #280 | Niger |
| #281 | nicaragua |
| #282 | New Caledonia |
| #283 | Antilles |
| #284 | Netherlands |
| #285 | Nepal |
| #286 | Namibia |
| #287 | Burma |
| #288 | Myanma |
| #289 | Myanmar |
| #290 | Mozambique |
| #291 | Ifni |
| #292 | Morocco |
| #293 | Montenegro |
| #294 | Mongolia |
| #295 | Moldovian |
| #296 | Moldovia |
| #297 | Moldova |
| #298 | Middle East |
| #299 | Micronesia |
| #300 | Mexico |
| #301 | Melanesia |
| #302 | "Agalega Islands" |
| #303 | Mauritius |
| #304 | Mauritania |
| #305 | Marshall Islands |
| #306 | Malta Schema: syn |
| #307 | Malta |
| #308 | Mali |
| #309 | Nyasaland |
| #310 | Malawi |
| #311 | Sarawak |
| #312 | Sabah |
| #313 | Malay |
| #314 | Malaya |
| #315 | Malaysia |
| #316 | Malagasy Republic |
| #317 | Madagascar |
| #318 | Macedonia |
| #319 | Lithuania Schema: syn |
| #320 | Libya |
| #321 | Liberia |
| #322 | Basutoland |
| #323 | Lesotho |
| #324 | Lebanon |
| #325 | Latvia |
| #326 | Laos |
| #327 | "Lao PDR" |
| #328 | Kirgizstan |
| #329 | kirghizstan |
| #330 | Kirghiz |
| #331 | Kyrgyz Republic |
| #332 | Kirghizia |
| #333 | Kyrgyzstan |
| #334 | Kosovo |
| #335 | Korea |
| #336 | Kiribati |
| #337 | Kenya |
| #338 | Kazakh |
| #339 | Kazakhstan |
| #340 | Jordon |
| #341 | Jamaica |
| #342 | Isle of Man |
| #343 | Iraq |
| #344 | Iran |
| #345 | Indonesia |
| #346 | Maldives |
| #347 | India |
| #348 | Hungary |
| #349 | Honduras |
| #350 | Haiti |
| #351 | Guyana |
| #352 | guiana guyana |
| #353 | Guiana Guyaya |
| #354 | Guiana |
| #355 | Guam |
| #356 | Guinea |
| #357 | Guatemala |
| #358 | Guatemela |
| #359 | guatemala |
| #360 | Grenada |
| #361 | Greece |
| #362 | Gold Coast |
| #363 | Ghana |
| #364 | Georgian Republic |
| #365 | Georgia Republic |
| #366 | Gaza |
| #367 | Gambia |
| #368 | Gabonese Republic |
| #369 | Gabon |
| #370 | Fiji |
| #371 | Ethiopia |
| #372 | Estonia |
| #373 | Eritrea |
| #374 | El Salvador |
| #375 | United Arab Republic |
| #376 | Egypt |
| #377 | Ecuador |
| #378 | Timor Leste |
| #379 | East Timur |
| #380 | East Timor |
| #381 | dominican republic |
| #382 | Dominician Republic |
| #383 | Dominica |
| #384 | dominici |
| #385 | Dominicia |
| #386 | French Somaliland |
| #387 | Djibouti |
| #388 | Slovak Republic |
| #389 | Slovakia |
| #390 | Czech Republic |
| #391 | Czechoslovakia |
| #392 | czechoslovakia |
| #393 | Cyprus |
| #394 | Cuba |
| #395 | Croatia |
| #396 | cote d'ivoire Ivory Coast |
| #397 | cote d'ivoire |
| #398 | Costa Rica |
| #399 | Zaire |
| #400 | Congo |
| #401 | Mayotte |
| #402 | Comoro Islands |
| #403 | comoro islands |
| #404 | Comores |
| #405 | Comoros |
| #406 | Colombia |
| #407 | China |
| #408 | Chile |
| #409 | Chad |
| #410 | Central African Republic |
| #411 | Cape Verde |
| #412 | Camerons |
| #413 | Cameron |
| #414 | Cameroons |
| #415 | Cameroon |
| #416 | Kampuchea |
| #417 | Khmer Republic |
| #418 | Cambodia |
| #419 | Urundi |
| #420 | Burundi |
| #421 | Upper Volta |
| #422 | Burkina Fasso |
| #423 | Burkina Faso |
| #424 | Bulgaria |
| #425 | Brazil |
| #426 | Brasil |
| #427 | Botswana |
| #428 | Herzegovina |
| #429 | Bosnia |
| #430 | Bolivia |
| #431 | Bhutan |
| #432 | Belize |
| #433 | Belorussia |
| #434 | Belorussian |
| #435 | Belarus |
| #436 | Byelorussian |
| #437 | Byelarus |
| #438 | Benin |
| #439 | Barbados |
| #440 | Bangladesh |
| #441 | Bahrain |
| #442 | Azerbaijan |
| #443 | Aruba |
| #444 | Armenian |
| #445 | Armenia |
| #446 | Argentina |
| #447 | Barbuda |
| #448 | Antigua |
| #449 | Angola |
| #450 | Algeria |
| #451 | Albania |
| #452 | "Afghanistan" |
| #453 | "Eastern Europe" |
| #454 | "Pacific Islands" |
| #455 | "Commonwealth of Independent States" |
| #456 | "Atlantic Islands" |
| #457 | Central America |
| #458 | Latin America |
| #459 | South America |
| #460 | "West Indies" |
| #461 | "Caribbean" |
| #462 | "Asia" |
| #463 | "Africa" |
| #464 | "transitional country" |
| #465 | "transitional countries" Schema: syn |
| #466 | "lami countries" |
| #467 | "lami country" |
| #468 | Lmics |
| #469 | Lmic |
| #470 | "third world" |
| #471 | "lower income economy" |
| #472 | "low income economy" |
| #473 | " lower income economies" |
| #474 | " low income economies" |
| #475 | " middle income economies" |
| #476 | " middle income economy" |
| #477 | " underdeveloped economy" |
| #478 | " underdeveloped economies" |
| #479 | " under developed economies" |
| #480 | " under developed economy" |
| #481 | " lesser developed economy" |
| #482 | " lesser developed economies" |
| #483 | " less developed economies" |
| #484 | " less developed economy" |
| #485 | "developing economies" |
| #486 | developing economy |
| #487 | "poorer world" |
| #488 | "poorer populations" |
| #489 | "poorer population" |
| #490 | "poorer countries" |
| #491 | "poorer country" |
| #492 | "poorer nations" |
| #493 | poorer nation |
| #494 | "poor world" |
| #495 | "poor population" |
| #496 | "poor populations" |
| #497 | "poor nations" |
| #498 | "poor nation" |
| #499 | "poor countries" |
| #500 | "poor country" |
| #501 | "deprived world" |
| #502 | "deprived populations" |
| #503 | "deprived population" |
| #504 | "deprived nation" |
| #505 | "deprived nations" |
| #506 | "deprived countries" |
| #507 | "deprived country" |
| #508 | "under served world" |
| #509 | "underserved world" |
| #510 | "underserved countries" |
| #511 | "underserved country" |
| #512 | "under served nations" |
| #513 | "under served nation" |
| #514 | "underserved nation" |
| #515 | "underserved nations" |
| #516 | "underserved population" |
| #517 | "underserved populations" |
| #518 | "under served populations" |
| #519 | under served population |
| #520 | "under served countries" |
| #521 | "under served country" |
| #522 | "low income countries" |
| #523 | "low income country" |
| #524 | "low income population" |
| #525 | "low income populations" |
| #526 | "low income nations" |
| #527 | low income country |
| #528 | "middle income populations" |
| #529 | "middle income population" |
| #530 | "middle income nations" |
| #531 | "middle income nation" |
| #532 | "middle income countries" |
| #533 | "middle income country" |
| #534 | "underdeveloped world" |
| #535 | "underdeveloped populations" |
| #536 | "underdeveloped population" |
| #537 | "underdeveloped nations" |
| #538 | "underdeveloped nation" |
| #539 | "underdeveloped nation |
| #540 | underdeveloped nation |
| #541 | underdeveloped nation |
| #542 | under developed world |
| #543 | under developed nation |
| #544 | under developed countries |
| #545 | under developed country |
| #546 | under developed populations |
| #547 | under developed population |
| #548 | under developed nations |
| #549 | lesser developed world |
| #550 | lesser developed population |
| #551 | lesser developed nations |
| #552 | lesser developed nation |
| #553 | lesser developed countries |
| #554 | lesser developed country |
| #555 | less developed world |
| #556 | less developed populations |
| #557 | less developed population |
| #558 | less developed nations |
| #559 | less developed nation |
| #560 | less developed countries |
| #561 | less developed country |
| #562 | developing world |
| #563 | developing populations |
| #564 | developing population |
| #565 | developing nations |
| #566 | developing nation |
| #567 | developing countries |
| #568 | developing country |
| #569 | (("Afghanistan" OR "Africa" OR "Agalega Islands" OR "Asia" OR "Atlantic Islands" OR "Caribbean" OR "Commonwealth of Independent States" OR "Eastern Europe" OR "Lao PDR" OR "Pacific Islands" OR "West Indies" OR "Albania" OR "Algeria" OR "Angola" OR "Antigua" OR "Antilles" OR "Argentina" OR "Armenia" OR "Armenian" OR "Aruba" OR "Azerbaijan" OR "Bahrain" OR "Bangladesh" OR "Barbados" OR "Barbuda" OR "Basutoland" OR "Belarus" OR "Belize" OR "Belorussia" OR "Belorussian" OR "Benin" OR "Bhutan" OR "Bolivia" OR "Bosnia" OR "Botswana" OR "Brasil" OR "Brazil" OR "Bulgaria" OR "Burkina Faso" OR "Burkina Fasso" OR "Burma" OR "Burundi" OR "Byelarus" OR "Byelorussian" OR "Cambodia" OR "Cameron" OR "Camerons" OR "Cameroon" OR "Cameroons" OR "Cape Verde" OR "Central African Republic" OR "Central America" OR "Ceylon" OR "Chad" OR "Chile" OR "China" OR "Colombia" OR "Comores" OR "Comoro Islands" OR "comoro islands" OR "Comoros" OR "Congo" OR "Costa Rica" OR "cote d'ivoire" OR "cote d'ivoire Ivory Coast" OR "Croatia" OR "Cuba" OR "Cyprus" OR "Czech Republic" OR "Czechoslovakia" OR "czechoslovakia" OR "Djibouti" OR "Dominica" OR "dominican republic" OR "dominici" OR "Dominicia" OR "Dominician Republic" OR "East Timor" OR "East Timur" OR "Ecuador" OR "Egypt" OR "El Salvador" OR "Eritrea" OR "Estonia" OR "Ethiopia" OR "Fiji" OR "French Somaliland" OR "Gabon" OR "Gabonese Republic" OR "Gambia" OR "Gaza" OR "Georgia Republic" OR "Georgian Republic" OR "Ghana" OR "Gold Coast" OR "Greece" OR "Grenada" OR "Grenadines" OR "Guam" OR "Guatemala" OR "guatemala" OR "Guatemela" OR "Guiana" OR "guiana guyana" OR "Guiana Guyaya" OR "Guinea" OR "Guyana" OR "Haiti" OR "Hebrides" OR "Herzegovina" OR "Honduras" OR "Hungary" OR "Ifni" OR "India" OR "Indonesia" OR "Iran" OR "Iraq" OR "Isle of Man" OR "Jamaica" OR "Jordon" OR "Kampuchea" OR "Kazakh" OR "Kazakhstan" OR "Kenya" OR "Khmer Republic" OR "Kirghiz" OR "Kirghizia" OR "kirghizstan" OR "Kirgizstan" OR "Kiribati" OR "Korea" OR "Kosovo" OR "Kyrgyz Republic" OR "Kyrgyzstan" OR "Laos" OR "Latin America" OR "Latvia" OR "Lebanon" OR "Lesotho" OR "Liberia" OR "Libya" OR "Lithuania Schema: syn" OR "Macedonia" OR "Madagascar" OR "Malagasy Republic" OR "Malawi" OR "Malay" OR "Malaya" OR "Malaysia" OR "Maldives" OR "Mali" OR "Malta" OR "Malta Schema: syn" OR "Marshall Islands" OR "Mauritania" OR "Mauritius" OR "Mayotte" OR "Melanesia" OR "Mexico" OR "Micronesia" OR "Middle East" OR "Moldova" OR "Moldovia" OR "Moldovian" OR "Mongolia" OR "Montenegro" OR "Montenegro" OR "Morocco" OR "Mozambique" OR "Muscat" OR "Myanma" OR "Myanmar" OR "Namibia" OR "Navigator Island" OR "Navigator Islands" OR "Nepal" OR "Netherlands" OR "Nevis" OR "New Caledonia" OR "New Hebrides" OR "nicaragua" OR "Niger" OR "Nigeria" OR "Northern Mariana Islands" OR "Nyasaland" OR "Oman" OR "Pakistan" OR "Palau" OR "Palestine" OR "Panama" OR "Paraguay" OR "Peru" OR "Philipine" OR "Philipines" OR "Philippine" OR "Philippines" OR "Poland" OR "Portugal" OR "Puerto Rico" OR "Rhodesia " OR "Romania" OR "Roumania" OR "Ruanda" OR "Rumania" OR "Russia" OR "Russian" OR "Rwanda" OR "Sabah" OR "Saint Kitts" OR "Saint Lucia" OR "Saint Vincent" OR "Samoa" OR "Samoan Islands" OR "Sao Tome" OR "Sarawak" OR "Saudi Arabia" OR "Senegal" OR "Serbia" OR "Seychelles" OR "Sierra Leone" OR "Slovak Republic" OR "Slovakia" OR "Slovenia" OR "Solomon Islands" OR "Somalia" OR "South America" OR "Soviet Union" OR "Sri lanka" OR "St Kitts" OR "St Lucia" OR "St Vincent" OR "Sudan" OR "Surinam" OR "Suriname" OR "Swaziland" OR "Syria" OR "Syrian" OR "tadzhik" OR "Tadzhikistan" OR "Tajikistan" OR "Tanzania" OR "Thailand" OR "Timor Leste" OR "Tobago" OR "Togo" OR "Togolese Republic" OR "Tonga" OR "Trinidad" OR "Tunisia" OR "Turkey" OR "Turkmen" OR "Turkmenistan" OR "Tuvalu" OR "Uganda" OR "Ukraine" OR "United Arab Republic" OR "Upper Volta" OR "Uruguay" OR "Urundi" OR "USSR" OR "Uzbek" OR "Uzbekistan" OR "Vanuatu" OR "Venezuela" OR "Viet Nam" OR "Vietnam" OR "West Bank" OR "Yemen" OR "Yugoslavia" OR "Zaire" OR "Zambia" OR "Zimbabwe" OR "Eastern Europe" OR "Pacific Islands" OR "Commonwealth of Independent States" OR "Atlantic Islands" OR "Central America" OR "Latin America" OR "South America" OR "West Indies" OR "Caribbean" OR "Asia" OR "Africa" OR "transitional country" OR "transitional countries Schema: syn" OR "lami countries" OR "lami country" OR "lmics" OR "lmic" OR "third world" OR "lower income economy" OR "low income economy" OR "lower income economies" OR "low income economies" OR "middle income economies" OR "middle income economy" OR "underdeveloped economy" OR "underdeveloped economies" OR "under developed economies" OR "under developed economy" OR "lesser developed economy" OR "lesser developed economies" OR "less developed economies" OR "less developed economy" OR "developing economies" OR "developing economy" OR "poorer world" OR "poorer populations" OR "poorer population" OR "poorer countries" OR "poorer country" OR "poorer nations" OR "poorer nation" OR "poor world" OR "poor population" OR "poor populations" OR "poor nations" OR "poor nation" OR "poor countries" OR "poor country" OR "deprived world" OR "deprived populations" OR "deprived population" OR "deprived nation" OR "deprived nations" OR "deprived countries" OR "deprived country" OR "under served world" OR "underserved world" OR "underserved countries" OR "underserved country" OR "under served nations" OR "under served nation" OR "underserved nation" OR "underserved nations" OR "underserved population" OR "underserved populations" OR "under served populations" OR "under served population" OR "under served countries" OR "under served country" OR "low income countries" OR "low income country" OR "low income population" OR "low income populations" OR "low income nations" OR " low income country" OR "middle income populations" OR "middle income population" OR "middle income nations" OR "middle income nation" OR "middle income countries" OR "middle income country" OR "underdeveloped world" OR "underdeveloped populations" OR "underdeveloped population" OR "underdeveloped nations" OR "underdeveloped nation" OR "underdeveloped nation" OR "underdeveloped nation" OR "underdevelopednation" OR "under developed world" OR "under developed nation" OR "under developed countries" OR "under developed country" OR "under developed populations" OR "under developed population" OR "under developed nations" OR "lesser developed world" OR "lesser developed population" OR "lesser developed nations" OR "lesser developed nation" OR "lesser developed countries" OR "lesser developed country" OR "less developed world" OR "less developed populations" OR "less developed population" OR "less developed nations" OR "less developed nation" OR "less developed countries" OR "less developed country" OR "developing world" OR "developing populations" OR "developing population" OR "developing nations" OR "developing nation" OR "developing countries" OR " developing country")) |
| #570 | Violence |
| #571 | suicide based[Title] |
| #572 | opioid[Title] |
| #573 | injur* Schema: syn |
| #574 | eating disorder[Title] |
| #575 | anxiety[Title] |
| #576 | attention deficit hyperactivity disorder[Title] |
| #577 | Adhd |
| #578 | Depression |
| #579 | disability[Title/Abstract] |
| #580 | Disability |
| #581 | Chronic obstructive Pulmonary Disease[Title] |
| #582 | chronic hepatitis[Title] |
| #583 | Alzheimer[Title] |
| #584 | Parkinson* |
| #585 | skin/ |
| #586 | immun* |
| #587 | Malaria |
| #588 | Maternal |
| #589 | Falls |
| #590 | Older |
| #591 | Children |
| #592 | Child |
| #593 | human immunodeficiency virus |
| #594 | HIV |
| #595 | Tuberculosis |
| #596 | Vaccines |
| #597 | HPV |
| #598 | Hepatitis |
| #599 | autism Schema: syn |
| #600 | Schizophrenia |
| #601 | (((((((((((((((((((((((((((((violence) OR suicide based[Title]) OR opioid[Title]) OR eating disorder[Title]) OR anxiety[Title]) OR attention deficit hyperactivity disorder[Title]) OR adhd) OR depression) OR disability[Title/Abstract]) OR disability) OR Chronic obstructive Pulmonary Disease[Title]) OR chronic hepatitis[Title]) OR Alzheimer[Title]) OR Parkinson*) OR skin/) OR immun*) OR malaria) OR maternal) OR falls) OR older) OR children) OR child) OR human immunodeficiency virus) OR HIV) OR tuberculosis) OR Vaccines) OR HPV) OR hepatitis) OR schizophrenia |
| #602 | 81 AND 146 AND 174 AND 569 |
| #603 | # 602 NOT 601 |
| #604 | [Filters activated: Randomized Controlled Trial, Duplicate Publication, Books and Documents, Publication date from 2000/01/01 to 2015/10/21, Humans. Clear all to show 130 items.](http://www.ncbi.nlm.nih.gov/pubmed) |

Embase

'accredited social health activist' OR 'activists' OR 'agents communitaria de salud' OR 'agents communitaria de saude' OR 'aide' OR'aides' OR 'allied health personnel'/exp OR 'allied health personnel' OR 'allied health worker' OR 'anganwadi' OR 'animatrice' OR'asha' OR 'assistant' OR 'attendant' OR 'auxiliary' OR 'barefoot doctor'/exp OR 'barefoot doctor' OR 'basic health worker' OR'brigadistas' OR 'caregiver'/exp OR 'caregiver' OR 'carer'/exp OR 'carer' OR 'chws' OR 'colaborador voluntary' OR 'colaborador voluntary schema: all' OR 'community'/exp OR 'community' OR 'community drug distributors' OR 'community health agent' OR'community health care workers' OR 'community health workers'/exp OR 'community health workers' OR 'community healthcare workers' OR 'community member' OR 'community resource persons' OR 'community worker' OR 'consultant'/exp OR 'consultant' OR'distributor' OR 'health auxiliary'/exp OR 'health auxiliary' OR 'health extension worker' OR 'health extension workers' OR 'health visitor'/exp OR 'health visitor' OR 'health visitors' OR 'health volunteer' OR 'health worker'/exp OR 'health worker' OR 'helper' OR'home health aide' OR 'lady health worker' OR 'lady health workers' OR 'lay' OR 'lay volunteer' OR 'lhws' OR 'line workers' OR 'link worker' OR 'midwife'/exp OR 'midwife' OR 'midwives'/exp OR 'midwives' OR 'monitor'/exp OR 'monitor' OR 'mother coordinator' OR'nonprofessional' OR 'nonprofessional workers' OR 'outreach educator' OR 'person' OR 'promotora' OR 'raise' OR 'rural health motivator' OR 'seika' OR 'shasthyo karmi' OR 'shasthyo karmi schema: all' OR 'shasthyo shebika' OR 'staff'/exp OR 'staff' OR'support worker' OR 'surveyor' OR 'trained' OR 'unlicensed' OR 'untrained' OR 'vhws' OR 'village drug-kit manager' OR 'village health helpers' OR 'visit' OR 'visitor' OR 'voluntary' OR 'voluntary worker'/exp OR 'voluntary worker' OR 'voluntary workers'/exp OR'voluntary workers' OR 'volunteer'/exp OR 'volunteer' OR 'volunteers'/exp OR 'volunteers' AND [randomized controlled trial]/lim AND ([article]/lim OR [conference abstract]/lim OR [conference paper]/lim) AND [humans]/lim AND [2000-2015]/py

'non communicable' OR 'non communicable disease' OR 'noncommunicable disease' OR 'noncommunicable' OR 'ncd' OR 'chronic disease' OR 'risk factors' OR 'cancer' OR 'cardiovascular disease' OR 'cvd' OR 'cvd risk' OR 'hypertension' OR 'life style factors' OR'ncds' OR 'cardiovascular diseases' OR 'raised blood pressure' OR 'diabetes' OR 'diabetes mellitus' OR 'cholesterol' OR'cholesterolemia' OR 'raised cholesterol' OR 'high triglycerides' OR 'triglycerides' OR 'body mass index' OR 'bmi' OR 'raised bmi' OR'overweight' OR 'obesity' OR 'obese' OR 'waist circumference' OR 'alcohol drinking' OR 'drinking' OR 'tobacco' OR 'tobacco smoke'OR diet OR 'nutrition' OR 'food habits' OR 'fruit' OR 'fruits' OR 'fruits and vegetables' OR 'vegetables' OR 'vegetable consumption' OR'vegetable intake' OR '5 a day' OR 'five a day' OR 'less than five servings' OR 'physical inactivity' OR 'exercise' OR 'regular exercise'OR 'smoking/exp' OR 'smoking behaviour' OR 'alcohol' OR 'alcohol consumption' OR 'stress/exp' OR 'running' OR 'jogging' OR'walking' OR 'walk' OR 'junk food' OR 'fast food'

'multiple risk behavior program' OR 'multiple risk behaviour program' OR 'multiple risk behavior intervention' OR 'multiple risk behaviour intervention' OR 'multiple behaviour risk factor program' OR 'multiple behavior risk factor program' OR 'multiple behaviour risk factor intervention' OR 'multiple behavior risk factor intervention' OR 'multiple health behaviour change intervention' OR 'multiple health behavior change intervention' OR 'health behaviour intervention' OR 'health behaviour program'OR 'health behavior intervention' OR 'health behavior program' OR 'multifactorial lifestyle program' OR 'multifactorial lifestyle intervention' OR 'multiple risk factor intervention' OR 'multiple risk factor program' OR 'behaviour change program' OR 'behaviour change intervention' OR 'behavior change program' OR 'behavior change intervention' OR 'life style intervention' OR 'lifestyle programs' OR 'lifestyle program' OR 'lifestyle interventions' OR 'lifestyle intervention'

'afghanistan' OR 'agalega islands' OR 'lao pdr' OR 'albania' OR 'algeria' OR 'angola' OR 'antigua' OR 'antilles' OR 'argentina' OR'armenia' OR 'armenian' OR 'aruba' OR 'azerbaijan' OR 'bahrain' OR 'bangladesh' OR 'barbados' OR 'barbuda' OR 'basutoland' OR'belarus' OR 'belize' OR 'belorussia' OR 'belorussian' OR 'benin' OR 'bhutan' OR 'bolivia' OR 'bosnia' OR 'botswana' OR 'brasil' OR'brazil' OR 'bulgaria' OR 'burkina faso' OR 'burkina fasso' OR 'burma' OR 'burundi' OR 'byelarus' OR 'byelorussian' OR 'cambodia' OR'cameron' OR 'camerons' OR 'cameroon' OR 'cameroons' OR 'cape verde' OR 'central african republic' OR 'ceylon' OR 'chad' OR 'chile'OR 'china' OR 'colombia' OR 'comores' OR 'comoro islands' OR 'comoros' OR 'congo' OR 'costa rica' OR 'cote d ivoire' OR 'cote d ivoire ivory coast' OR 'croatia' OR 'cuba' OR 'cyprus' OR 'czech republic' OR 'czechoslovakia' OR 'djibouti' OR 'dominica' OR 'dominican republic' OR 'dominici' OR 'dominicia' OR 'dominician republic' OR 'east timor' OR 'east timur' OR 'ecuador' OR 'egypt' OR 'el salvador' OR 'eritrea' OR 'estonia' OR 'ethiopia' OR 'fiji' OR 'french somaliland' OR 'gabon' OR 'gabonese republic' OR 'gambia' OR'gaza' OR 'georgia republic' OR 'georgian republic' OR 'ghana' OR 'gold coast' OR 'greece' OR 'grenada' OR 'grenadines' OR 'guam' OR'guatemala' OR 'guatemela' OR 'guiana' OR 'guiana guyana' OR 'guiana guyaya' OR 'guinea' OR 'guyana' OR 'haiti' OR 'hebrides' OR'herzegovina' OR 'honduras' OR 'hungary' OR 'ifni' OR 'india' OR 'indonesia' OR 'iran' OR 'iraq' OR 'isle of man' OR 'jamaica' OR'jordon' OR 'kampuchea' OR 'kazakh' OR 'kazakhstan' OR 'kenya' OR 'khmer republic' OR 'kirghiz' OR 'kirghizia' OR 'kirghizstan' OR'kirgizstan' OR 'kiribati' OR 'korea' OR 'kosovo' OR 'kyrgyz republic' OR 'kyrgyzstan' OR 'laos' OR 'latvia' OR 'lebanon' OR 'lesotho' OR'liberia' OR 'libya' OR 'lithuania schema: syn' OR 'macedonia' OR 'madagascar' OR 'malagasy republic' OR 'malawi' OR 'malay' OR'malaya' OR 'malaysia' OR 'maldives' OR 'mali' OR 'malta' OR 'malta schema: syn' OR 'marshall islands' OR 'mauritania' OR'mauritius' OR 'mayotte' OR 'melanesia' OR 'mexico' OR 'micronesia' OR 'middle east' OR 'moldova' OR 'moldovia' OR 'moldovian' OR'mongolia' OR 'montenegro' OR 'morocco' OR 'mozambique' OR 'muscat' OR 'myanma' OR 'myanmar' OR 'namibia' OR 'navigator island' OR 'navigator islands' OR 'nepal' OR 'netherlands' OR 'nevis' OR 'new caledonia' OR 'new hebrides' OR 'nicaragua' OR 'niger'OR 'nigeria' OR 'northern mariana islands' OR 'nyasaland' OR 'oman' OR 'pakistan' OR 'palau' OR 'palestine' OR 'panama' OR'paraguay' OR 'peru' OR 'philipine' OR 'philipines' OR 'philippine' OR 'philippines' OR 'poland' OR 'portugal' OR 'puerto rico' OR'rhodesia' OR 'romania' OR 'roumania' OR 'ruanda' OR 'rumania' OR 'russia' OR 'russian' OR 'rwanda' OR 'sabah' OR 'saint kitts' OR'saint lucia' OR 'saint vincent' OR 'samoa' OR 'samoan islands' OR 'sao tome' OR 'sarawak' OR 'saudi arabia' OR 'senegal' OR 'serbia'OR 'seychelles' OR 'sierra leone' OR 'slovak republic' OR 'slovakia' OR 'slovenia' OR 'solomon islands' OR 'somalia' OR 'soviet union'OR 'sri lanka' OR 'st kitts' OR 'st lucia' OR 'st vincent' OR 'sudan' OR 'surinam' OR 'suriname' OR 'swaziland' OR 'syria' OR 'syrian' OR'tadzhik' OR 'tadzhikistan' OR 'tajikistan' OR 'tanzania' OR 'thailand' OR 'timor leste' OR 'tobago' OR 'togo' OR 'togolese republic' OR'tonga' OR 'trinidad' OR 'tunisia' OR 'turkey' OR 'turkmen' OR 'turkmenistan' OR 'tuvalu' OR 'uganda' OR 'ukraine' OR 'united arab republic' OR 'upper volta' OR 'uruguay' OR 'urundi' OR 'ussr' OR 'uzbek' OR 'uzbekistan' OR 'vanuatu' OR 'venezuela' OR 'viet nam'OR 'vietnam' OR 'west bank' OR 'yemen' OR 'yugoslavia' OR 'zaire' OR 'zambia' OR 'zimbabwe' OR 'eastern europe' OR 'pacific islands' OR 'commonwealth of independent states' OR 'atlantic islands' OR 'central america' OR 'latin america' OR 'south america'OR 'west indies' OR 'caribbean' OR 'asia' OR 'africa' OR 'transitional country' OR 'lami countries' OR 'lami country' OR 'lmics' OR'lmic' OR 'third world' OR 'lower income economy' OR 'low income economy' OR 'lower income economies' OR 'low income economies' OR 'middle income economies' OR 'middle income economy' OR 'underdeveloped economy' OR 'underdeveloped economies' OR 'under developed economies' OR 'under developed economy' OR 'lesser developed economy' OR 'lesser developed economies' OR 'less developed economies' OR 'less developed economy' OR 'developing economies' OR 'developing economy' OR 'poorer world' OR 'poorer populations' OR 'poorer population' OR 'poorer countries' OR 'poorer country' OR 'poorer nations' OR 'poorer nation' OR 'poor world' OR 'poor population' OR 'poor populations' OR 'poor nations' OR 'poor nation' OR 'poor countries' OR 'poor country' OR 'deprived world' OR 'deprived populations' OR 'deprived population' OR 'deprived nation' OR 'deprived nations' OR 'deprived countries' OR 'deprived country' OR 'under served world' OR 'underserved world' OR 'underserved countries' OR 'underserved country' OR 'under served nations' OR 'under served nation' OR 'underserved nation' OR 'underserved nations' OR 'underserved population' OR 'underserved populations' OR 'under served populations' OR 'under served population' OR 'under served countries' OR 'under served country' OR 'low income countries' OR 'low income population' OR 'low income populations' OR 'low income nations' OR 'low income country' OR 'middle income populations' OR 'middle income population' OR 'middle income nations' OR 'middle income nation' OR 'middle income countries' OR 'middle income country' OR 'underdeveloped world' OR 'underdeveloped populations' OR 'underdeveloped population' OR 'underdeveloped nations' OR 'underdeveloped nation' OR 'under developed world' OR 'under developed nation' OR 'under developed countries' OR 'under developed country' OR 'under developed populations' OR 'under developed population' OR 'under developed nations' OR 'lesser developed world' OR 'lesser developed population' OR 'lesser developed nations' OR 'lesser developed nation' OR 'lesser developed countries' OR 'lesser developed country' OR 'less developed world' OR 'less developed populations' OR 'less developed population' OR 'less developed nations' OR 'less developed nation' OR 'less developed countries' OR 'less developed country' OR 'developing world' OR 'developing populations 'OR 'developing population' OR 'developing nations' OR 'developing nation' OR 'developing countries' OR 'developing country'

'randomised controlled trial' OR 'randomized controlled trial' OR 'randomized' OR 'randomised' OR 'cluster randomised trial' OR 'intervention' OR 'interventions' OR 'cluster randomisation' OR 'cluster randomization' OR cluster AND randomized OR 'group randomized trial' OR 'group randomized' OR 'group randomised' OR 'control' OR 'controlled' OR 'controlled trial'

'violence' OR 'suicide based' OR 'opioid' OR 'eating disorder' OR 'anxiety' OR 'attention deficit hyperactivity disorder' OR 'adhd' OR 'depression' OR 'disability' OR 'chronic obstructive pulmonary disease' OR 'chronic hepatitis' OR 'alzheimer' OR 'parkinsonism' OR'skin' OR 'malaria' OR 'pregnant' OR 'falls' OR 'older' OR 'children' OR 'child' OR 'human immunodeficiency virus' OR 'hiv' OR'tuberculosis' OR 'vaccines' OR 'hpv' OR 'hepatitis' OR 'schizophrenia'
