# Supplementary material for: Community health workers for non-communicable diseases prevention and control in developing countries: Evidence and implications
Source: PLoS One. 2017 Jul 13;12(7):e0180640. doi: 10.1371/journal.pone.0180640 (PMC5509237; doi:10.1371/journal.pone.0180640)

S2 Text: Meta-Analysis Details

1. Combining intervention arm: For one trial which had 3 intervention arms, 2 of the arms were combined as the interventions were in the same intensity category. We used the following formulae to calculate combined means and standard deviations.

Mean change from baseline (M combined) for combined groups:

(N_1_ M_1_+N_2_M_2_)/ (N_1_+N_2_)

Standard deviation of mean change from baseline (SD combined) for combining


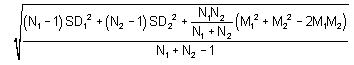


1. We used standard calculations to convert 95% Confidence Intervals to Standard Deviation.

Formula for converting 95% CI to SD


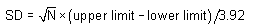


1. The effect of an intervention on a continuous outcome is measured by the absolute difference between the mean outcome observed for the experimental intervention and control, termed the mean difference (MD). This estimates the amount by which the treatment changes the outcome on average and is expressed:


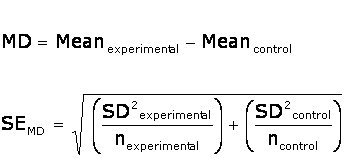

Supplement: S2 Text — (DOCX) [file pone.0180640.s003.docx]
