# Supplementary material for: Community health workers for non-communicable diseases prevention and control in developing countries: Evidence and implications
Source: PLoS One. 2017 Jul 13;12(7):e0180640. doi: 10.1371/journal.pone.0180640 (PMC5509237; doi:10.1371/journal.pone.0180640)
Supplement: S1 Table — (DOCX) [file pone.0180640.s004.docx]

S1 Table: Community Health Worker Characteristics

| Study Id | Name given in article | Training | Supervision | Ratio of CHW to participants |
| --- | --- | --- | --- | --- |
| De Pue JD, 2013 | CHW | Yes | Nurse practitioner | 2:1 |
| Hasandokht T, 2015 | Nurse | Yes | None described | 1:40 |
| Jafar TH, 2015 | CHW | Yes | None described | 1:1 |
| Mash RJ, 2014 | Health Promoters | Yes | Independent researchers using standardised tools | 1:15 |
| Mohlman MK, 2013 | *Raedat Refeyat* | Yes | Independent researchers using standardised tools | Unclear, but group intervention |
| Pazoki R, 2007 | Health Promoters (Healthy Heart Trainer group | Yes, 4 workshops | Advisory Board of Community members | 1:5 |
| Garcia-Pena C, 2002 | Nurse | Yes | Not Clear | 1:1 |
| Thankappan KR, 2013 | Non-doctor health professional | Yes, with examination and certification | None described | 1:1 |
| Joshi R, 2013 | Non Physician Health Worker | Yes | Physicians recorded his decisions alongside those of NPHW | Unclear |
| Jayakrishnan R, 2013 | Female Community Health Workers | Yes | Active monitoring of collected Data | 1:1 |
| Mendis S, 2010 (C) | Health Worker | Yes, 3- week structured course | None described | 1:1 |
| Mendis S, 2010 (N) | Health Worker | Yes, every 2-4 months | None described | 1:1 |
| Lee LL, 2006 | Public Health Nurse | Yes | None described | 1:1 |
| Goldhaber-Fiebert JD, 2003 | Nurse Educators, walking group leaders (volunteers) | Yes | None described | 1:15 |
| Zhong X, 2015 | Peer leaders | Yes | Monitoring through research team records and work report forms of CHSC | 1:15 |
| Wattana, 2007 | Registered Nurse | Yes | Clinical Committee of community hospitals | Group session/ Group Discussion ratio not mentioned, Home visits 1:1 |
| Cappucio FP, 2006 | Community Health Workers | Yes | None described | Not recorded due to large numbers |
