# Supplementary material for: Community health workers for non-communicable diseases prevention and control in developing countries: Evidence and implications
Source: PLoS One. 2017 Jul 13;12(7):e0180640. doi: 10.1371/journal.pone.0180640 (PMC5509237; doi:10.1371/journal.pone.0180640)
Supplement: S2 Table — (DOCX) [file pone.0180640.s005.docx]

S2 Table: Intervention Contents in detail

| Study Id | Theoretical basis (with key references) | Primary Purpose | Content | Main Format(s) used by community Health Worker | Additional Formats | Source |
| --- | --- | --- | --- | --- | --- | --- |
| De Pue JD, 2013 | Proceed Precede model, Chronic Care Model | Disease Management by treatment and Primary Prevention of NCDs | Intervention content was guided by both patient risk and self-selected goals from a menu of the following eight topics: diabetes introduction; healthy eating; being active; using medication; monitoring (understanding and using information from BG and BP measurement, tracking progress); reducing risk (preventing complications, standards of care, visits and laboratory measurements, smoking, alcohol, foot care); healthy coping (managing stress and depression); and problem- solving. | Print Media | None | Four Steps to control your life |
| Hasandokht T, 2015 | None Mentioned | Disease Management by treatment and Primary Prevention of NCDs | The intervention was provided over the course of four sessions weekly. Each session had a theme incorporating the specific domains of a lifestyle modification program concluded with a 15 to 20-min guided relaxation. Participants received written manual and a CD-ROM containing PowerPoint slides (Microsoft office 2007) in movie format covered in each of the four sessions for their review and relaxation music for home practice. Nurses working in these health centres were trained about lifestyle modification package by the researcher. | health Education material and weekly health messages | Text message | JNC 7, DASH, AHA guidelines for adults, |
| Jafar TH, 2010 | ? | Prevention | Screening visit: The Community Health Workers (CHW) will pay home visits to invite all subjects aged 5 years or over to participate in the survey. The screening would have three levels: 1) Household screening for subjects aged 5 years and over (n= 17,850 individuals, 3000 households). 2) Adult screening for individuals aged 40 years or over (n= 4200 individuals). 3) Hypertensive adults screening (n= 1860 individuals) for those identified to have hypertension on adult screening will be invited for re-measurement of BP to confirm hypertension. | Print media | None | 7th report of JNC, fourth working party of the British Hypertension Society Guidelines |
| Mash RJ, 2014 | Motivational interviewing using elicit provide elicit approach | Preventive | understanding Diabetes, living a healthy lifestyle, understanding the medication, understanding complications | Print Medial | SMS reminders | Conversation map |
| Mohlman MK, 2013 | None described as such. | Preventive | Guide booklet, pamphlet on smoking and passive smoking hazards, CDs with lectures on smoking hazards wherein researchers focussed upon supplementing Egypt's "top down" regulatory approach to tobacco control with a "bottom up" strategy of outreach and education for households. | Direct Verbal Communication | NC | Not mentioned |
| Pazoki R, 2007 | None described as such. | Preventive | Audio-taped activity instructions with music and practical usage of the educational package (4 booklets and weekly newsletters) were given to the intervention group in weekly home-visits by 53 volunteers from local non-governmental and community-based organizations. | Print media/ Audio | Audio taped instructions | Choose to Move Program |
| Garcia-Pena C, 2002 | NC | Prevention and Disease Management | During home visits, the nurse measured blood pressure and reviewed information from the baseline health check, and discussed possible lifestyle changes. The nurses tried to guide their patients to a healthier lifestyle and suggested different alternative ways to achieve the changes and negotiated speciﬁc targets. | Direct Communication | None | Not mentioned |
| Thankappan KR, 2013 | Not mentioned | Prevention | Counselling using the five “As” (Ask, Assess, Advise, Assist, Arrange) and the five “Rs” (Relevance, Risks, Rewards, Roadblocks, Repetition) | Not mentioned | Educational Material | Not mentioned |
| Joshi R, 2013 | Not mentioned | Prevention | Health Promotion Campaigns including posters, rallies, community presentations | Mass media | 0 | Not mentioned |
| Jayakrishnan R, 2013 | Behaviour change wheel’ model, | Cessation | Advice which can range from simple verbal instructions to quit smoking to the extent of providing information on the harmful effects of smoking in detail. | Print media and Direct contact | None | Quick reference guide for tobacco cessation titled “How to quit tobacco?” [Developed by the Tobacco Cessation Clinic, Regional Cancer Centre (RCC), Thiruvananthapuram in the local language, (Malayalam)] |
| Mendis S, 2010 (C) | Not mentioned | Prevention of Cardiovascular Diseases | The study included four visits: baseline, 4 months, 8 months and 12 months. The cost of visits was not covered. At the baseline visit all subjects at intervention sites were counselled on risk factor control (tobacco cessation, diet, physical activity), but no drug treatment was given. Patient information materials, translated to the local language, were given to each subject, along with follow-up cards to encourage adherence. The cards documented blood pressure measurements and indicated future visit dates. The back of the cards contained a summary of the counselling on diet, tobacco cessation and physical activity. At the second visit (at 4 months), subjects were classified into low and medium CVD risk categories | Direct contact as well as print media | None | CVD risk management protocol for the primary care level (scenario 1 of the WHO CVD risk management package) |
| Lee LL, 2006 | Self efficacy | Preventive | Advice for regular walking with benefits of increased walking, overcoming perceived barriers, sharing information on pleasant walking routes, use of pedometer and completing walking logs | Direct contact | None | American College of Sports Medicine's physical activity guidelines |
| Goldhaber-Fiebert JD, 2003 | Not mentioned | Disease Management by treatment and Primary Prevention of NCDs | 11 weekly nutrition classes (90 minutes each) and walking group | Direct Contact | Food diaries, measuring cups for portion control, hand outs | Specially designed curriculum of local customs |
| Zhong X, 2015 | Not mentioned | Prevention and Disease Management | 12 Biweekly 1.5 to 2 hours educational meetings and Biweekly discussion meetings | Direct Contact | Health promotion activities (e.g., walking and tai chi groups) and informal individual support through casual contact | None described |
| Wattana, 2007 | self-efficacy and self-management theories | Prevention and Disease Management | One group diabetes education class, 4 small group discussions, 2 individual home visits sessions | Direct Contact | None | Patient Education manual, "Living well with Diabetes |
| Cappucio FP, 2006 | Not Mentioned | Prevention | 24 weekly educational and health promotion sessions | Direct Contact | None | Standard Health Education Package from Ghana Ministry of Health |
