# Supplementary material for: Community health workers for non-communicable diseases prevention and control in developing countries: Evidence and implications
Source: PLoS One. 2017 Jul 13;12(7):e0180640. doi: 10.1371/journal.pone.0180640 (PMC5509237; doi:10.1371/journal.pone.0180640)
Supplement: S3 Table — (DOCX) [file pone.0180640.s006.docx]

S3 Table: Characteristics of Excluded studies and Studies awaiting Classification

| Excluded study | Reason for exclusion |
| --- | --- |
| Allen et al, 2011 | Secondary Prevention |
| Daivadanam M, 2013 | Study awaiting classification |
| Pengpid S et al , 2014 | Trial not started |
| Sathish T et al , 2013 | Study awaiting classification |
| Weber MB et al, 2012 | Study awaiting classification |
| Wei X et al, 2013 | Study awaiting classification |
| Yan LL et al, 2014 | Study awaiting classification |
| Aung MN et al, 2013 | Study awaiting classification |
| Mittra I et al, 2009 | Secondary Prevention |
| Dinshaw K et al, 2007 | Secondary Prevention |
| Shastri SS et al, 2014 | Secondary Prevention |
| Sankaranarayanan et al, 2013 | Secondary Prevention |
| Sankaranarayanan et al, 2005 | Secondary Prevention |
| Pisani P et al, 2006 | Secondary Prevention |
| Sankaranarayanan R et al, 2007 | Secondary Prevention |
| Acik y et al, 2005 | Non randomised controlled trial |
