# Supplementary material for: Community health workers for non-communicable diseases prevention and control in developing countries: Evidence and implications
Source: PLoS One. 2017 Jul 13;12(7):e0180640. doi: 10.1371/journal.pone.0180640 (PMC5509237; doi:10.1371/journal.pone.0180640)
Supplement: S4 Table — (DOCX) [file pone.0180640.s007.docx]

S4 Table: Matrix Highlighting Multicomponent Nature of Interventions for NCD control

|  | Tobacco | Body weight | BMI | Blood Pressure | Waist circumference | Waist- hip ratio | Body fat % | Total energy intake | Total fat intake | Saturated fat intake | % energy fat | Fruit intake | Vegetable intake | Fibre intake | Physical Activity (Moderate) | Physical Activity (Vigorous) | Fasting Blood Sugar | Total Cholesterol | Sodium intake | Stress |
| --- | --- | --- | --- | --- | --- | --- | --- | --- | --- | --- | --- | --- | --- | --- | --- | --- | --- | --- | --- | --- |
| De Pue JD, 2013 | + | + | + | 0 | + | 0 | 0 | 0 | + | 0 | 0 | 0 | 0 | 0 | + | 0 | 0 | 0 | 0 | 0 |
| Hasandokht T, 2015 | + | + | + | + | + | 0 | 0 | + | 0 | 0 | 0 | + | + | 0 | + | + | 0 | 0 | + | + |
| Jafar TH, 2015 | + | + | + | + | 0 | + | 0 | 0 | + | + | 0 | + | + | 0 | + | + | 0 | 0 | + | 0 |
| Mash RJ, 2014 | 0 | + | 0 | 0 | + | 0 | 0 | 0 | 0 | 0 | 0 | 0 | 0 | 0 | 0 | 0 | 0 | 0 | 0 | 0 |
| Mohlman MK, 2013 | + | 0 | 0 | 0 | 0 | 0 | 0 | 0 | 0 | 0 | 0 | 0 | 0 | 0 | 0 | 0 | 0 | 0 | 0 | 0 |
| Pazoki R, 2007 | + | + | + | + | 0 | + | 0 | 0 | 0 | 0 | 0 | 0 | 0 | 0 | + | + | + | + | 0 | 0 |
| Garcia-Pena C, 2002 | 0 | + | 0 | + | 0 | 0 | 0 | 0 | 0 | 0 | 0 | + | + | 0 | + | + | 0 | 0 | 0 | 0 |
| Thankappan KR, 2013 | + | 0 | 0 | 0 | 0 | 0 | 0 | 0 | 0 | 0 | 0 | 0 | 0 | 0 | 0 | 0 | 0 | 0 | 0 | 0 |
| Joshi R, 2013 | + | 0 | 0 | 0 | 0 | 0 | 0 | 0 | 0 | 0 | 0 | + | + | + | + | + | 0 | 0 | 0 | 0 |
| Jayakrishnan R, 2013 | + | 0 | 0 | 0 | 0 | 0 | 0 | 0 | 0 | 0 | 0 | 0 | 0 | 0 | 0 | 0 | 0 | 0 | 0 | 0 |
| Mendis S, 2010 (C) | + | 0 | + | + | 0 | 0 | 0 | 0 | 0 | 0 | 0 | 0 | + | + | 0 | 0 | 0 | 0 | 0 | 0 |
| Mendis S, 2010 (N) | 0 | 0 | 0 | 0 | 0 | 0 | 0 | 0 | 0 | 0 | 0 | 0 | 0 | 0 | 0 | 0 | 0 | 0 | 0 | 0 |
| Lee LL, 2006 | 0 | 0 | 0 | + | 0 | 0 | 0 | 0 | 0 | 0 | 0 | 0 | 0 | + | 0 | 0 | 0 | 0 | 0 | 0 |
| Goldhaber-Fiebert JD, 2003 | 0 | + | + | + | 0 | 0 | 0 | 0 | + | + | 0 | + | + | + | + | 0 | + | + | 0 | 0 |
| Zhong X, 2015 | 0 | 0 | 0 | 0 | 0 | 0 | 0 | 0 | 0 | 0 | 0 | + | + | + | + | + | 0 | 0 | 0 | + |
| Wattana, 2007 | 0 | 0 | 0 | 0 | 0 | 0 | 0 | 0 | 0 | 0 | 0 | 0 | 0 | 0 | 0 | 0 | 0 | 0 | 0 | 0 |
| Cappucio FP, 2006 | 0 | 0 | 0 | + | 0 | 0 | 0 | 0 | 0 | 0 | 0 | 0 | 0 | 0 | 0 | 0 | 0 | 0 | + | 0 |
| Studies addressing the factor | 9 | 7 | 6 | 8 | 3 | 2 | 0 | 1 | 3 | 2 | 0 | 6 | 7 | 5 | 8 | 6 | 2 | 2 | 3 | 2 |
| % studies | 56 | 44 | 38 | 50 | 19 | 13 | 0 | 6 | 19 | 13 | 0 | 38 | 44 | 31 | 50 | 38 | 13 | 13 | 19 | 13 |
